# Quantile regression analysis reveals widespread evidence for gene-environment or gene-gene interactions in myopia development

**DOI:** 10.1038/s42003-019-0387-5

**Published:** 2019-05-06

**Authors:** Alfred Pozarickij, Cathy Williams, Pirro G. Hysi, Jeremy A. Guggenheim, UK Biobank Eye and Vision Consortium, Tariq Aslam, Sarah A. Barman, Jenny H. Barrett, Paul Bishop, Peter Blows, Catey Bunce, Roxana O. Carare, Usha Chakravarthy, Michelle Chan, Sharon Y.L. Chua, David P. Crabb, Philippa M. Cumberland, Alexander Day, Parul Desai, Bal Dhillon, Andrew D. Dick, Cathy Egan, Sarah Ennis, Paul Foster, Marcus Fruttiger, John E.J. Gallacher, David F. Garway-Heath, Jane Gibson, Dan Gore, Chris J. Hammond, Alison Hardcastle, Simon P. Harding, Ruth E. Hogg, Pearse A. Keane, Sir Peng T. Khaw, Anthony P. Khawaja, Gerassimos Lascaratos, Andrew J. Lotery, Tom Mac Gillivray, Sarah Mackie, Keith Martin, Michelle McGaughey, Bernadette McGuinness, Gareth J. McKay, Martin McKibbin, Danny Mitry, Tony Moore, James E. Morgan, Zaynah A. Muthy, Eoin O’Sullivan, Chris G. Owen, Praveen Patel, Euan Paterson, Tunde Peto, Axel Petzold, Jugnoo S. Rahi, Alicja R. Rudnikca, Jay Self, Sobha Sivaprasad, David Steel, Irene Stratton, Nicholas Strouthidis, Cathie Sudlow, Dhanes Thomas, Emanuele Trucco, Adnan Tufail, Veronique Vitart, Stephen A. Vernon, Ananth C. Viswanathan, Katie Williams, Jayne V. Woodside, Max M. Yates, Jennifer Yip, Yalin Zheng

**Affiliations:** 1 School of Optometry & Vision Sciences, Cardiff University, Cardiff, CF24 4HQ UK; 2 Population Health Sciences, Bristol Medical School, University of Bristol, Bristol, BS8 2BN UK; 3 Department of Ophthalmology, King’s College London, St. Thomas’ Hospital, London, SE1 7EH UK; 4 Department of Twin & Genetic Epidemiology, King’s College London, St. Thomas’ Hospital, London, SE1 7EH UK; 5 University of Manchester, Manchester, UK; 6 Kingston University, Kingston, UK; 7 University of Leeds, Leeds, UK; 8 NIHR Biomedical Research Centre, London, UK; 9 King’s College London, London, UK; 10 University of Southampton, Southampton, UK; 11 Queens University Belfast, Belfast, UK; 12 University College London, London, UK; 13 University of Edinburgh, Edinburgh, UK; 14 University of Bristol, Bristol, UK; 15 University of Oxford, Oxford, UK; 16 University of Liverpool, Liverpool, UK; 17 University of Cambridge, Cambridge, UK; 18 Leeds Teaching Hospitals NHS Trust, Leeds, UK; 19 Cardiff University, Cardiff, UK; 20 King’s College Hospital NHS Foundation Trust, London, UK; 21 University of London, London, UK; 22 Newcastle University, Tyne, UK; 23 Gloucestershire Hospitals NHS Foundation Trust, Cheltenham, UK; 24 University of Dundee, Dundee, UK; 25 Nottingham University Hospitals NHS Trust, Nottingham, UK; 26 University of East Anglia, Norwich, UK

**Keywords:** Genetic interaction, Refractive errors

## Abstract

A genetic contribution to refractive error has been confirmed by the discovery of more than 150 associated variants in genome-wide association studies (GWAS). Environmental factors such as education and time outdoors also demonstrate strong associations. Currently however, the extent of gene-environment or gene-gene interactions in myopia is unknown. We tested the hypothesis that refractive error-associated variants exhibit effect size heterogeneity, a hallmark feature of genetic interactions. Of 146 variants tested, evidence of non-uniform, non-linear effects were observed for 66 (45%) at Bonferroni-corrected significance (*P* < 1.1 × 10^−4^) and 128 (88%) at nominal significance (*P* < 0.05). *LAMA2* variant rs12193446, for example, had an effect size varying from −0.20 diopters (95% CI −0.18 to −0.23) to −0.89 diopters (95% CI −0.71 to −1.07) in different individuals. SNP effects were strongest at the phenotype extremes and weaker in emmetropes. A parsimonious explanation for these findings is that gene-environment or gene-gene interactions in myopia are pervasive.

## Abstract (web summary)

Alfred Pozarickij et al. present an analysis of effect-size heterogeneity among myopia-associated variants in the UK Biobank dataset. They find evidence for non-uniform effects for 45% of variants using a conservative cutoff, suggesting that gene-environment and/or gene-gene interactions are pervasive in myopia.

## Introduction

The prevalence of refractive error has doubled in several parts of the world in the past few decades^1–3^. By 2050 it is predicted that 50% of the world population will be myopic (near-sighted), with 4.8 billion individuals affected^4^. Myopia is associated with axial elongation of the eye, which increases the risk of retinal detachment, myopic maculopathy, glaucoma, and other pathological complications, making it an increasingly common cause of visual impairment and blindness^5–7^. Susceptibility to myopia is determined both by genetic and environmental factors. Genome-wide association studies (GWAS) have identified ~150 genetic variants associated with refractive error^8–11^, including some overlap with monogenic disease gene loci^12^. The time children spend outdoors, time performing near-viewing tasks, and the number of years in education are also strongly associated with myopia development^13–20^.

In conventional GWAS analyses of quantitative traits, it is assumed that each copy of a genetic variant shifts the phenotype by the same amount in all individuals, i.e. genetic effect sizes are assumed to be uniform. This assumption feeds forward into metrics such as SNP-heritability, and polygenic risk scores (PRS) used for genetic prediction. However, loci with gene-gene (GxG) or gene-environment (GxE) interactions will violate this assumption: for these loci the (marginal) effect size of a variant varies from person to person, depending on their genotype at other loci or their environmental exposure profile (for variants involved in GxG and GxE interactions, respectively). Accordingly, a number of elegant studies have used evidence of a non-uniform effect size across individuals as a ‘signature’ to identify GxG or GxE interaction loci^21–24^. A major advantage of this approach is that it does not require the identity of the environmental risk factor underlying a GxE effect to be pre-specified or measured, nor the identity of the second genetic variant to be known when detecting GxG interactions. Instead, the presence of GxG or GxE interaction can be inferred using only genotype information for a genetic marker and phenotype information for the trait of interest.

Since GxE effects are implicated in myopia susceptibility^25–28^, and yet currently very few such interacting variants have been discovered, we aimed to comprehensively assess the known genetic variants associated with refractive error for involvement in interactions by testing for this ‘signature’ of non-uniform genetic effect sizes across individuals. We compared our results for refractive error with those for height, a highly polygenic trait with little or no evidence of gene-environment or gene-gene interactions.

## Results

In the sample of 72,985 unrelated, European-ancestry participants whose genotype data passed quality control and had phenotype information available, the mean ± SD refractive error was −0.25 ± 2.67 diopters (D) and the average age was 57.8 ± 7.8 years.

We assessed 146 genetic variants that showed genome-wide significant association (*p* < 5 × 10^−8^) with refractive error in a recent meta-analysis carried out by the CREAM Consortium and 23andMe and that showed evidence of independent replication in the UK Biobank sample^11^. We coded the risk allele as the allele associated with a more negative refractive error.

### Conventional ordinary least squares (OLS) analysis

A standard, ordinary least squares (OLS) linear regression analysis of SNP effects under the assumption of constant effect size across all individuals produced very similar results to those reported previously in UK Biobank participants^11^ (Supplementary Data 1). Of the 146 variants tested, the strongest effect was for rs12193446 near *LAMA2*, which was associated with a −0.43 D more negative refractive error (95% CI from −0.39 to −0.48, *p* = 1.1 × 10^−77^).

### Conditional quantile regression and meta-regression (CQR-MR)

**Fig. 1:**
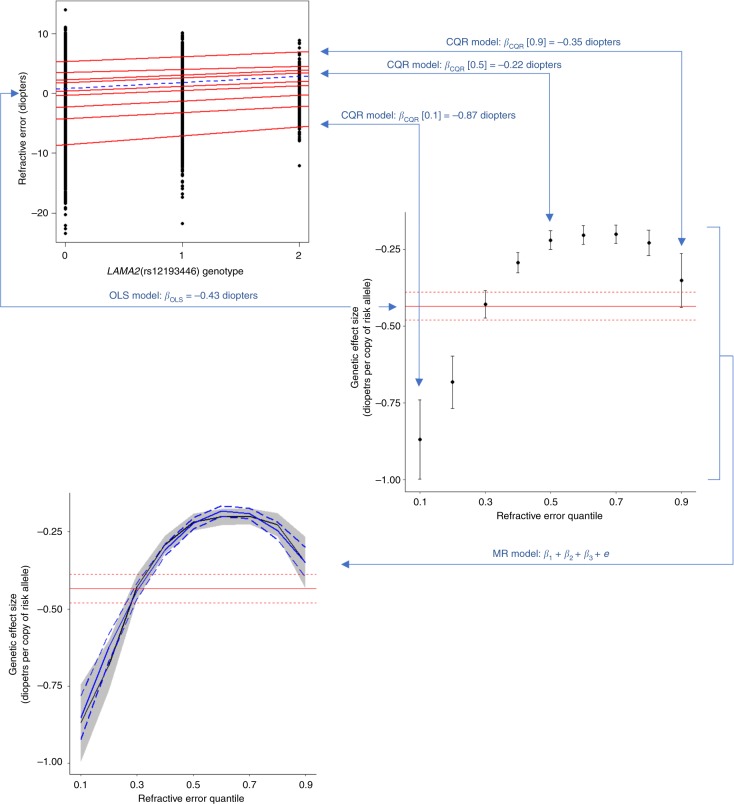
Conditional quantile regression (CQR) and meta-regression (MR) can identify if genetic effect size varies in individuals depending on their position in the trait distribution. In conventional ordinary least squares (OLS) linear regression, SNP effect size is estimated under the assumption that it is the same for every person in the sample. Thus, the effect size is calculated as the slope of the regression line (dashed blue line in top-left graph) obtained by minimizing the sum of squared residuals between data points and the mean, for each genotype class (0, 1 or 2 copies of the minor allele). Alternatively, in CQR, the SNP effect size is estimated at a specific quantile of the outcome distribution. Analogous to OLS, the effect size is calculated as the slope of the quantile regression line (in the top-left graph, the nine red lines correspond to quantile regression fits for quantiles 0.1, 0.2, 0.3, …, 0.9 of the trait distribution). For the variant shown, rs12193446, the effect size (slope) differs for individuals in different quantiles of the trait distribution; this can be visualized more readily by plotting the effect size at each quantile (black circles with error bars in middle-right graph). OLS analysis assumes the effect size is constant across quantiles of the trait distribution (horizontal red line in middle-right graph, with dotted red lines indicating 95% CI). After using CQR to estimate the SNP effect size at a range of quantiles, the uniformity of the SNP effect sizes can be quantitatively assessed using MR (solid blue line in bottom-left graph, with dashed blue lines showing 95% CI)

Figure 1 illustrates the CQR-MR analysis process, and contrasts it with OLS regression. Whereas an OLS model seeks to minimize the sum of squared residuals between data points and the mean effect for each genotype class (AA, AB, and BB), a quantile regression model seeks to minimize the absolute residuals at a specific quantile of trait distribution for each genotype class. Crucially, unlike OLS regression, CQR allows a variant’s genetic effect size to vary between individuals, depending on their position in the trait distribution (Fig. 1).

The type I error rate and statistical power of CQR-MR were investigated (see Methods) and full results are presented in the Supplementary Notes 1 and 2. The main finding was a systematic inflation of the type I error rate of CQR-MR that was independent of MAF (Supplementary Fig. 1), but that this could be readily corrected using a ‘genomic control’ approach. This correction was applied in all of the results presented below. The statistical power of CQR-MR varied depending on the number of different quantiles included in the meta-regression. The use of 9 quantiles spaced equally at 0.1 intervals was found to perform well (Supplementary Fig. 1) and hence was applied in all of the present analyses.

### Widespread evidence of non-uniform effects sizes

**Fig. 2:**
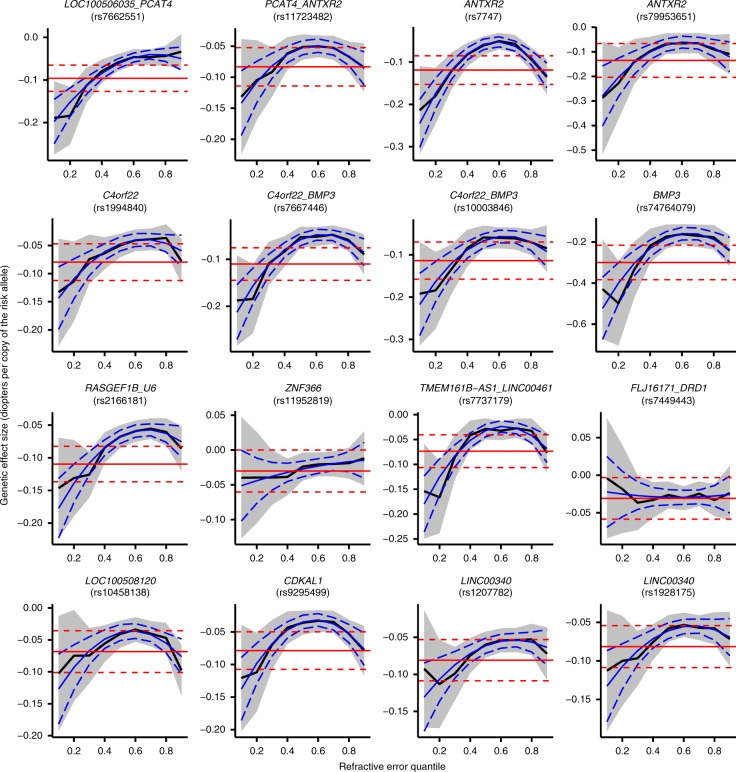
Changes in genetic effect size across the refractive error distribution for a representative subset of genetic variants associated with refractive error. Genetic effect size estimates from conditional quantile regression (CQR) are represented by the solid black line and their 95% confidence intervals are shown by the shaded grey region. The solid red line is the effect size estimate from conventional linear regression analysis with its 95% confidence intervals shown by the red dashed lines. Effect size estimates from meta-regression are shown with the solid blue line with corresponding 95% confidence intervals given by the dashed blue lines

CQR-MR was used to determine if effect sizes for the 146 refractive error-associated variants differed across individuals depending on their position (i.e. their quantile) in the refractive error distribution. Nearly all variants exhibited an inverse-U shaped effect size profile, with the strongest effect size in individuals at the extremes of the refractive error distribution and a minimum effect size in emmetropic participants near the center of the distribution. Representative results are presented in Fig. 2 (results for all variants are shown in Supplementary Fig. 2). For instance, for rs12193446 (*LAMA2*), which had the strongest effect in the conventional OLS analysis, the effect size varied from −0.20 D (95% CI from −0.18 to −0.23) for individuals near the centre of the trait distribution to −0.89 D (95% CI from −0.71 to −1.07) for the most highly myopic individuals (Fig. 1). Exceptions to the inverse-U shaped effect size pattern were observed for variants such as rs1649068 (*BICC1*) and rs9388766 (*L3MBTL3*), which displayed non-constant, yet nearly linear changes in effect size across quantiles of the refractive error distribution, along with SNPs such as rs9680365 (*GRIK1*) and rs7449443 (*FLJ16171-DRD1*), which had essentially flat effect size profiles similar to those obtained under the OLS assumption of a constant effect size in all individuals.

### Quantitative analysis of non-uniform effects

We used a 3-parameter model to quantify the non-uniformity of effect sizes (see Methods). After correcting for multiple-testing by applying a Bonferroni adjusted *p*-value threshold of 0.05/(3 × 146) = 1.1 × 10^−4^, a total of 66 (45%) of the variants showed significant non-uniform effects, i.e. *p* < 1.1 × 10^−4^ for the *β*_1_ (linear) or *β*_2_ (quadratic) model coefficients (Table 1 and Supplementary Data 2). Thus, 45% of the genetic variants showed statistically significant evidence of differing effect sizes depending where in the refractive error distribution an individual lay, suggestive of the variant’s involvement in either a gene-gene or gene-environment interaction. For the rs12193446 (*LAMA2*) variant, *p* = 2.12 × 10^−36^ for the *β*_1_ component, and *p* = 1.19 × 10^−30^ for the *β*_2_ component. Notably, only 18 (12%) of the variants failed to show at least nominal evidence of an interaction effect (i.e. *β*_1_ component and *β*_2_ component, *p* > 0.05).

**Table 1:** Summary statistics for the 10 strongest associations with refractive error based on conditional quantile regression-meta-regression (CQR-MR)

SNP	Gene(s)	*β*_0_		*β*_1_		*β*_2_	
		Beta [95% CI]	*P*	Beta [95% CI]	*P*	Beta [95% CI]	*P*
rs12193446	*BC035400_LAMA2*	−1.130 [−1.272; −0.988]	8.07 × 10^−55^	2.995 [2.529; 3.461]	2.12 × 10^−36^	−2.363 [−2.765; −1.961]	1.19 × 10^−30^
rs524952	*GOLGA8B_GJD2*	−0.673 [−0.758; −0.588]	4.83 × 10^−54^	1.797 [1.534; 2.06]	7.47 × 10^−41^	−1.417 [−1.634; −1.200]	1.68 × 10^−37^
rs7744813	*KCNQ5*	−0.543 [−0.631; −0.455]	7.24 × 10^−34^	1.402 [1.132; 1.672]	2.15 × 10^−24^	−1.092 [−1.314; −0.870]	5.75 × 10^−22^
rs11602008	*LRRC4C*	−0.669 [−0.79; −0.548]	2.60 × 10^−27^	1.612 [1.250; 1.974]	2.71 × 10^−18^	−1.131 [−1.421; −0.841]	2.25 × 10^−14^
rs1550094	*PRSS56*	−0.521 [−0.624; −0.418]	4.77 × 10^−23^	1.441 [1.118; 1.764]	2.08 × 10^−18^	−1.142 [−1.409; −0.875]	4.90 × 10^−17^
rs72621438	*SNORA51_CA8*	−0.441 [−0.530; −0.352]	2.06 × 10^−22^	1.089 [0.817; 1.361]	4.46 × 10^−15^	−0.821 [−1.044; −0.598]	5.85 × 10^−13^
rs2326823	*BC035400*	−0.680 [−0.830; −0.530]	6.17 × 10^−19^	1.815 [1.341; 2.289]	6.45 × 10^−14^	−1.429 [−1.831; −1.027]	3.09 × 10^−12^
rs10500355	*RBFOX1*	−0.400 [−0.490; −0.310]	3.63 × 10^−18^	1.011 [0.734; 1.288]	8.39 × 10^−13^	−0.775 [−1.003; −0.547]	2.76 × 10^−11^
rs6495367	*RASGRF1*	−0.374 [−0.459; −0.289]	7.17 × 10^−18^	1.009 [0.747; 1.271]	4.38 × 10^−14^	−0.833 [−1.049; −0.617]	3.89 × 10^−14^
rs2573210	*PRSS56*	−0.501 [−0.621; −0.381]	2.91 × 10^−16^	1.414 [1.037; 1.791]	1.94 × 10^−13^	−1.121 [−1.434; −0.808]	2.26 × 10^−12^

Confidence intervals and *p*-values have been corrected for the inflated type I error rate of CQR-MR

*SNP* single nucleotide polymorphism, *CHR* chromosome, *BP* base pair, *EA* effect allele, *β*_0_ meta-regression intercept effect size in diopters per copy of the risk allele, *β*_1_ and *β*_2_ meta-regression coefficients for the linear and quadratic terms, respectively, *CI* confidence interval

For comparison, an analogous set of analyses to those performed above were carried out for genome-wide significant variants associated with height. For height, only 6% of variants (nine out of 148) displayed at least nominal evidence of a non-uniform effect size (Supplementary Note 3, Supplementary Data 3 and 4, and Supplementary Fig. 2).

### Polygenic risk score interaction with educational attainment

**Fig. 3:**
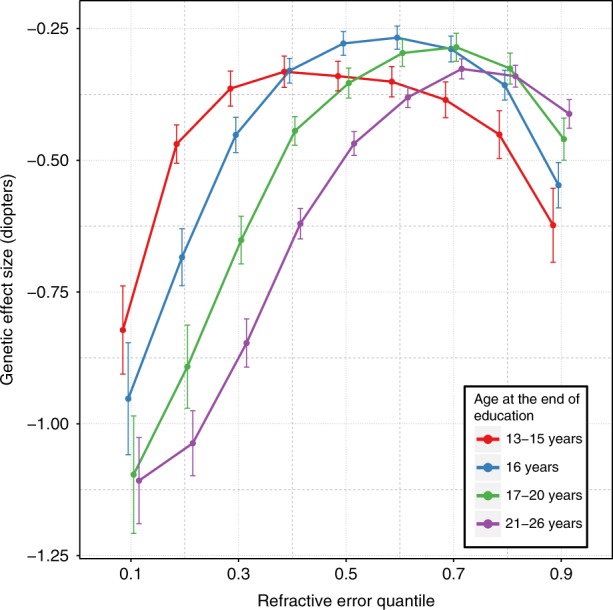
The effect of educational attainment on refractive error varies across quantiles of the refractive error distribution. Each line represents the polygenic risk score (PRS) effect size across quantiles for individuals with different times spent in education. Error bars show 95% confidence intervals

We used the 146 refractive error-associated variants to create a polygenic risk score (PRS) and examined whether this too exhibited a non-uniform effect size in different individuals. As shown in Fig. 3, the PRS effect size displayed the inverted-U pattern across quantiles of the trait distribution as was observed for the majority of individual SNPs. In addition, the PRS effect size differed across educational attainment strata. For participants from the myopic tail of the refractive error distribution, more time spent in education was associated with a larger PRS effect size. For example for those in refractive error quantile 0.1, a 1 SD increase in PRS was associated with a −0.82 D (95% CI from −0.73 to −0.90) more negative refractive error in the lowest educational stratum, yet a −1.11 D (95% CI from −1.02 to −1.18) more negative refractive error for those in the highest education stratum (*p* = 8.9 × 10^−83^ and *p* = 1.17 × 10^−155^, respectively). The largest change in PRS effect size due to such an interaction with education was 0.57 D (at quantile 0.2). The PRS effect size difference associated with educational attainment was smallest in emmetropes. For example, the PRS effect size was within a narrow range of −0.25 to −0.37 D for participants in quantile 0.6, irrespective of their level of education. For participants in the hyperopic tail of the refractive error distribution (quantiles > 0.8), the PRS effect size was smaller in those with greater educational attainment, opposite to the relationship seen in the myopic tail. Thus, for example, for hyperopic participants in quantile 0.9, a 1 SD reduction in PRS was associated with a +0.62 D (95% CI from +0.55 to +0.69) effect on refractive error in those in the lowest education stratum, yet only a +0.41 D (95% CI from +0.38 to +0.44) effect in those from the highest education stratum (*p* = 6.55 × 10^−68^ and *p* = 9.53 × 10^−193^, respectively).

## Discussion

Evidence of effect size heterogeneity—a signature of involvement in GxG or GxE interactions—was found for 88% of the refractive error-associated variants tested. Furthermore, the impact of this phenomenon was dramatic: genetic effect sizes were as much as four-fold higher in certain individuals compared to others. Previous studies of refractive error genetics have always assumed that genetic effect sizes are the same in every person in the sample, and thus this important source of inter-individual variation has remained hidden.

Refractive error-associated variants typically had inverse-U shaped effect size profiles, with the strongest effects observed at the phenotype extremes, and effects closer to zero in emmetropes. Very few SNPs had constant effects across all quantiles of the sample distribution that matched those assumed in conventional analyses. One potential explanation for these findings is the process of ‘emmetropization’, in which the rate of axial eye elongation during infancy is fine-tuned by a visual feedback loop in order to maintain a sharp retinal image^29^. We speculate that emmetropization may act as a buffer against the myopia- or hyperopia-predisposing effects of genetic risk variants. Thus, suppose that, during childhood, a myopia-predisposing risk allele led to a small increase in axial eye length. This might subsequently be countered by a slowing of the rate of axial elongation via visually-mediated feedback. Furthermore, suppose there exists a limit to the amount of axial elongation that the emmetropization system can compensate for (as has been proposed for the axial elongation-countering effects of crystalline lens thinning^30^) then in those individuals whose emmetropization limit is surpassed, genetic variants would have free reign to attain much higher effects than in those individuals whose emmetropization limit is not exceeded. Finding evidence to support a direct role for emmetropization in causing the observed genetic effect size heterogeneity of refractive error-associated variants would likely require studies in animal models; the recent discovery of a genetic locus for susceptibility to visually-induced myopia is a first step in this direction^31^.

Prior to this work, only a handful of specific GxE interactions, and no GxG interactions had been reported for refractive error^25–28^. The current work suggests that such interaction effects are likely to be widespread. Applying our same analysis methods to a different trait, height, yielded far fewer variants with signatures of a GxG or GxE interaction (6% for height vs. 88% for refractive error). Given that height and axial eye length share genetic determinants in common (genetic correlation 0.1–0.2)^32,33^, it would be interesting to examine genetic effect sizes across quantiles of the axial length distribution, for example in samples of emmetropes and myopes.

The PRS findings confirmed the dramatic difference in phenotypic effect exerted by refractive error-associated genetic variants in different individuals, which contrasts starkly with the simple deterministic effects expected of high risk genotypes. Individuals who reached adulthood as emmetropes appeared to have been ‘buffered’ against their genetic risk burden, and thus genetic effect sizes in these individuals were correspondingly small. By contrast, genetic effect sizes were often several-fold larger in individuals who became highly myopic or highly hyperopic by the time they reached adulthood. Time spent in education appeared to further modify the phenotypic effects of risk SNPs.

Our strategy for detecting inter-individual differences in genetic effect sizes was based on a statistical test for variance heterogeneity across genotypes. While variance heterogeneity is a signature of GxG and GxE interactions^21,34–36^, it is not the only cause. Parent-of-origin effects will give rise to increased variance heterogeneity in heterozygous individuals at loci in which the effect size varies dependent on which parent transmitted the risk allele^34^. Similarly, ‘genetic nurture’, whereby untransmitted alleles in parents (as well as transmitted alleles) influence the phenotype^37^ may also lead to variance heterogeneity. For example, if the environment of the child is partly determined by the parents’ genotype, then risk alleles inherited by the child will potentially show interactions with untransmitted parental alleles, i.e. an inter-generational GxG interaction mediated via a GxE interaction for the child. Allelic heterogeneity, whereby multiple genotypes in linkage disequilibrium influence the same phenotype, can also give rise to variance heterogenity^38–40^. Finally, examples of genetic variants with striking inter-individual genetic effect heterogeneity exist for which mechanistic explanations are currently lacking or incomplete. For instance, rs3825942 in *LOXL1* is associated with an increased risk of exfoliation syndrome in certain populations, but a reduced risk in others^41^ (so called risk allele ‘flipping’), and rs6817105 near *PITX2* is associated with an ~1.6-fold increased risk of atrial fibrillation overall; however, the level of risk varies widely across populations^42^. Explanations based on simple GxG or GxE interactions have not been able to account for the observed effect size heterogeneity at these loci^41,42^.

To conclude, our study provides evidence that most of the currently-known refractive error-associated variants have different effect sizes in different individuals. A parsimonious explanation is that the variants are involved in GxG or GxE interactions. The phenotypic effect imparted by risk alleles was found to vary as much as four-fold, with greater effects observed for individuals in the phenotype extremes compared to those in the center. This variation in inter-individual effects remains hidden when conventional analysis methods are used to detect genetic effects. Widespread GxG or GxE interactions will contribute to the ‘missing heritability’ for refractive error, and adversely impact the accuracy of genetic prediction of children at-risk of developing myopia.

## Methods

### Study participants and quality control

The UK Biobank project is an ongoing cohort study of ~500,000 UK adults aged 40–70-years-old when recruited (2006–2010)^43^. Ethical approval for the study was obtained from the National Health Service National Research Ethics Service (Ref 11/NW/0382) and all participants provided written informed consent. Participants provided a blood sample, from which DNA was extracted and genotyped using either the UK BiLEVE Axiom array or the UK Biobank Axiom Array^44^. We analysed data from the July 2016 data release for genetic variants in 488,377 individuals imputed to the HRC^45^ reference panel.

Participants self-reported whether they had a university or college degree. An ophthalmic assessment was introduced towards the latter stages of UK Biobank recruitment, hence only about 25% of participants were examined. Refractive error was measured using non-cycloplegic autorefraction (Tomey RC5000; Tomey GmbH Europe, Erlangen-Tennenlohe, Germany). The mean spherical equivalent (MSE) refractive error was calculated as the sphere power plus half the cylinder power, and averaged between the two eyes (*avMSE*). Individuals who self-reported any of the following eye disorders were excluded from the analyses: cataracts, “serious eye problems”, “eye trauma”, a history of cataract surgery, corneal graft surgery, laser eye surgery, or other eye surgery in the past 4 weeks. Individuals whose hospital records (ICD10 codes) indicated a history of the following were also excluded: cataract surgery, eye surgery, retinal surgery, or retinal detachment surgery. Of 488,377 individuals with genetic information, samples were excluded due to: ocular history (*n* = 48,145, see above), withdrawal of consent (*n* = 8), self-report of non-white British ethnicity or genetic principal components indicative of non-European ancestry (*n* = 69,938), outlying level of genetic heterozygosity (*n* = 648), or refractive error not measured (*n* = 283,352). The remaining 86,286 individuals were tested for relatedness using the --rel-cutoff command in PLINK v1.9^46^. A genetic relationship matrix was created using a linkage disequilibrium (LD)-pruned set of well-imputed variants (with IMPUTE2 *r*^*2*^ > 0.9, minor allele frequency (MAF) > 0.005, missing rate ≤ 0.01, and ‘rs’ variant ID prefix). LD-pruning was accomplished by using the --indep-pairwise 50 5 0.1 command in PLINK v2^46^. One member of each pair with genomic relatedness greater than 0.025 was excluded. This resulted in a final sample size of 72,985 unrelated individuals of European ancestry.

### Selection of genetic variants

#### Variants associated with refractive error

We originally assessed 149 genetic variants that showed genome-wide significant association (*p* < 5 × 10^−8^) with refractive error in the CREAM Consortium and 23andMe meta-analysis and that replicated in a UK Biobank sample^11^. The risk allele was coded as the allele associated with a more negative refractive error. Of the 149 genetic variants tested, reliable results could be obtained for 146 (for rs74764079, rs73730144, and rs17837871, with MAFs of 3%, 1% and 1%, respectively, there were fewer than 50 participants homozygous for the minor allele; hence these variants were excluded).

#### Variants associated with height

For comparison, we also examined genetic variants associated with height. GWAS summary statistics were obtained from Wood et al.^38^. We restricted our analyses to the 149 genetic variants with the strongest association (i.e. those with the lowest *p*-values). Reliable results could be obtained for 148 height SNPs (Supplementary Note 3).

### Statistical analysis

A ‘conventional’ OLS regression analysis was carried out to quantify the effect size of each of the 146 variants under the assumption of a constant effect size across the full sample. Refractive error averaged between the two eyes (*avMSE*) was the dependent variable and genotype, age, age-squared, sex, and a binary variable indicating genotyping array were fitted as covariates. Conditional quantile regression (CQR)^47^ was carried out using the *quantreg* package v5.36 in R version 3.5.1, using the same set of covariates as above. We used 10,000 Markov-chain-marginal-bootstrap replicates to calculate standard errors. As a sensitivity analysis, we also tested linear regression and quantile regression models with the first 10 principal components included as covariates. However, including principal components in the models did not change parameter estimates substantially, hence only the results of the original analyses are reported.

SNP effect estimates and their standard errors from quantile regression at 9 different quantiles (0.1, 0.2, 0.3, …, 0.9) were meta-regressed using a mixed-effects model (*metafor* package v2.0.0 in R^48^) with the estimated SNP effect at each quantile modelled as the dependent variable and the quantile at which these estimates were obtained as the independent variable. A term for quantile-squared was also included in the meta-regression model to test for non-linear genetic effects across quantiles, resulting in the model: *y* = *β*_0_ + *β*_1_*q* + *β*_2_*q*2 + *e* (where, *β*_0_ is an intercept term, *β*_1_ and *β*_2_ are coefficients describing the linear and quadratic change in SNP effect across quantiles of the trait distribution, respectively, *q* are the quantiles, and *e* is the error term). Figure 1 illustrates the conditional quantile regression and meta-regression model fitting strategy.

### Permutation-based assessment of type I error rate and power

To assess the type I error and power of the CQR-MR model we used the gold-standard method of permutation. The type 1 error rate was assessed in two ways. Firstly, we simulated genotypes for ‘null’ SNPs and tested for an association between the true phenotype and the null SNP genotype. Secondly, we permuted phenotype values amongst individuals in the sample, and tested for an association between the null phenotype and the observed (true) SNP genotypes.

*Null phenotype*: The *avMSE* phenotype of the 72,985 individuals in the analysis sample was permuted 100 times. For each permutation, the association between the null phenotype and the genotype of each of the 149 variants was assessed using CQR-MR. The type 1 error rate was calculated as the proportion of SNPs with *P* < 0.05 for each of the three meta-regression coefficients (*β*_0_, *β*_1_, and *β*_2_) from the total of (100 × 149) = 14,900 permutations. *Null SNPs:* The 72,985 individuals in our analysis sample were independently assigned genotypes for a biallelic SNP with MAF ranging from 0.05 to 0.45, simulated from a binomial distribution. Association between *avMSE* and the genotype of the null SNP was assessed using CQR-MR. The type 1 error rate was calculated as the proportion of SNPs with *P* < 0.05 for each of the three meta-regression coefficients (*β*_0_, *β*_1_, and *β*_2_) after simulating 10,000 null SNPs.

To obtain a relative indication of statistical power, the 149 refractive error-associated variants were tested for association with the observed *avMSE* phenotype in samples of varying size. Specifically, from the full sample of 72,985 individuals, we selected a random sample of 10,000–70,000 individuals, in steps of 10,000, and tested each of the 149 variants for association. This procedure was repeated 20 times. Power was computed as the proportion of replicates in which the null hypothesis was rejected at a nominal significance level of *α* = 0.05 (i.e. under the assumption that all 149 variants truly had non-linear effect sizes across quantiles). The total number of tests used for these power evaluations was 149 × 7 × 20 = 20,860. The same set of covariates as in original analysis was included in the CQR step when assessing power and type 1 error.

In the analyses described above, CQR-MR was performed by carrying out quantile regression at 9 different quantiles (*q* = 0.1 to 0.9 in steps of 0.1) followed by meta-regression of the resulting genetic effect size estimates. In preliminary work, we explored the effect on type 1 error rate and power of selecting more or fewer than 9 quantiles, by testing: (a) 19 quantiles, *q* = 0.05–0.95 in steps of 0.05; (b) 10 quantiles, *q* = 0.05–0.95 in steps of 0.1; (c) 5 quantiles, *q* = 0.1–0.9 in steps of 0.2. For simplicity, we refer to these CQR-MR models by the number of quantiles included in the meta-regression, i.e. 5, 9, 10, or 19. CQR-MR analysis with 9 quantiles performed optimally (Supplementary Notes and Supplementary Fig. 3).

### Gene-environment interaction with education

To test for the presence of gene-environment interaction, we constructed a polygenic risk score (PRS) by counting the number of risk alleles (0, 1, or 2) carried by each individual. We did not weight these by SNP effect sizes in order to avoid introducing bias by using weights obtained from, and applied in, the same sample (UK Biobank). ‘Age completed full-time education’ (*EduYears*) was selected as an exemplar environmental variable. UK Biobank participants with a university degree were not asked the age they completed full-time education, hence these individuals were assumed to have completed their education at the age of 21 years. Age completed education categories with low counts were merged with adjacent categories, resulting in four final *EduYears* categories: 13–15, 16, 17–20, and 21–26 years. We carried out a CQR-MR analysis stratified by *EduYears* category.

### Reporting summary

Further information on experimental design is available in the Nature Research Reporting Summary linked to this article.

## Supplementary information

Supplementary Information

Description of additional supplementary items

Supplementary Data

Reporting Summary

## Data Availability

Individual-level data from UK Biobank can be accessed by applying to the UK Biobank Central Access Committee (http://www.ukbiobank.ac.uk/register-apply/).

## Code availability

The R code for performing these analyses is available upon request.
