# Supplementary material for: Quantile regression analysis reveals widespread evidence for gene-environment or gene-gene interactions in myopia development
Source: Commun Biol. 2019 May 6;2:167. doi: 10.1038/s42003-019-0387-5 (PMC6502837; doi:10.1038/s42003-019-0387-5)
Supplement: Supplementary file 1 — Supplementary Information [file 42003_2019_387_MOESM1_ESM.pdf]

Supplementary Information

Quantile regression analysis reveals widespread evidence for gene-environment or gene-gene interactions in myopia development

Alfred Pozarickij, Cathy Williams, Pirro Hysi, Jeremy A. Guggenheim,
and the UK Biobank Eye and Vision Consortium.

Title	Page
Supplementary Figure 1. Type I error rate and power for CQR-MR models with different number of quantiles.	S2
Supplementary Figure 2. Changes in genetic effect size across the refractive error distribution for genetic variants associated with refractive error.	S4
Supplementary Figure 3. Changes in genetic effect size across the height distribution for genetic variants associated with height.	S13
Supplementary Note 1. Type I error and power of CQR-MR	S23
Supplementary Note 2. Adjustment for the inflated type 1 error rate of CQR-MR	S25
Supplementary Note 3. CQR-MR analysis of GIANT consortium variants associated with height	S26
Supplementary References	S27

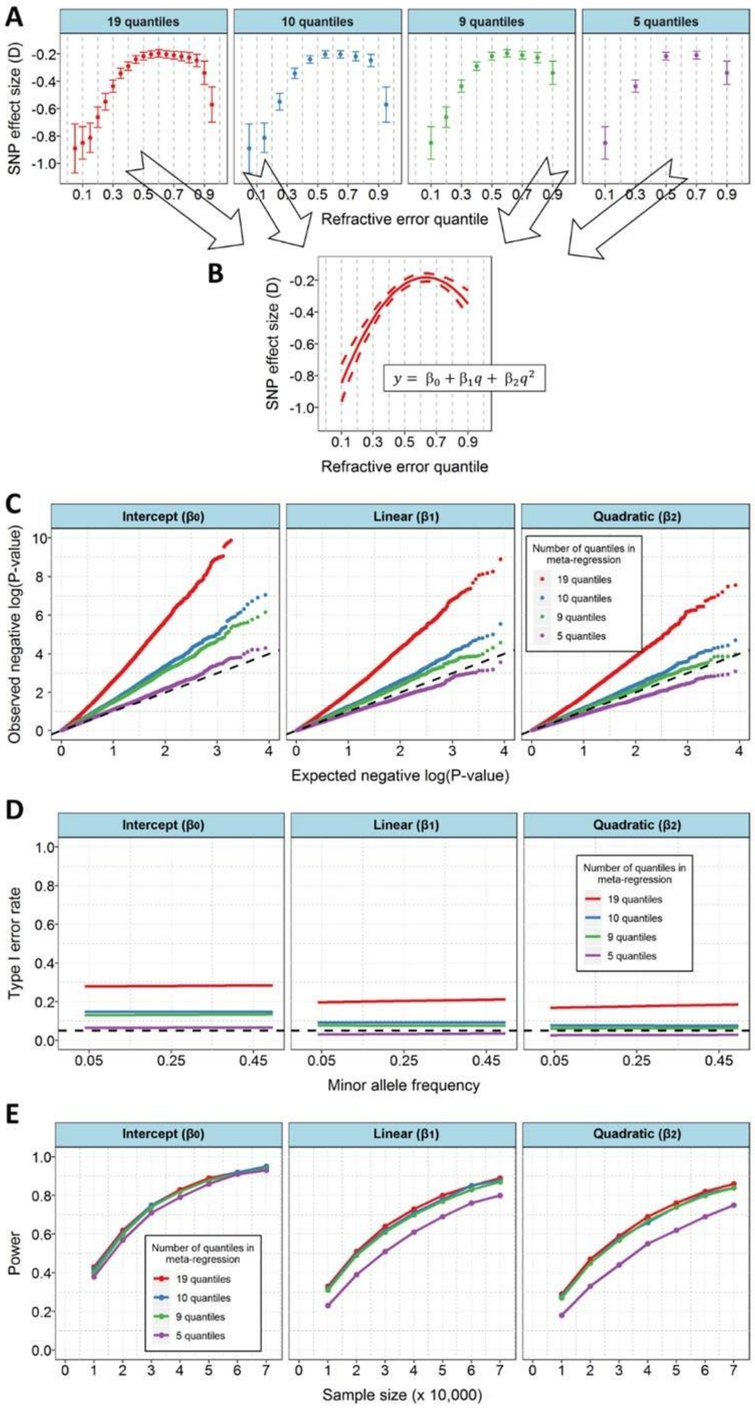

Supplementary Figure 1. Type I error rate and power for CQR-MR models with different number of quantiles. (A) Illustration of genetic effect size estimates for CREAM variant rs12193446 from CQR carried out at 19, 10, 9 or 5 quantiles across the trait distribution. (B) MR was used to fit a

quadratic function to the CQR results, in order to quantify the degree of non-uniformity and non-linearity of the CQR genetic effect size estimates. This yielded three coefficients describing the fit: intercept term β_0, linear term β_1, and quadratic term β_2. (C) CQR-MR models were fit for 14,900 'null phenotype' permutations. QQ-plots for observed versus expected p-values demonstrate systematic inflated test statistics (an excessive of low p-values) for MR models that included 19, 10 or 9 quantiles. The dashed black line is the line of unity. (D) The type I error rate for the models fit in (C). The dashed black line shows the correct type I error rate. Note the observed type I error rate is inflated for MR models including 19, 10 or 9 quantiles, and conservative for the 5 quantile MR model. (E) Relative statistical power of MR models including 19, 10, 9 or 5 quantiles, after adjusting for the inflated type I error rate. Note that power is lower for the 5 quantile MR model.

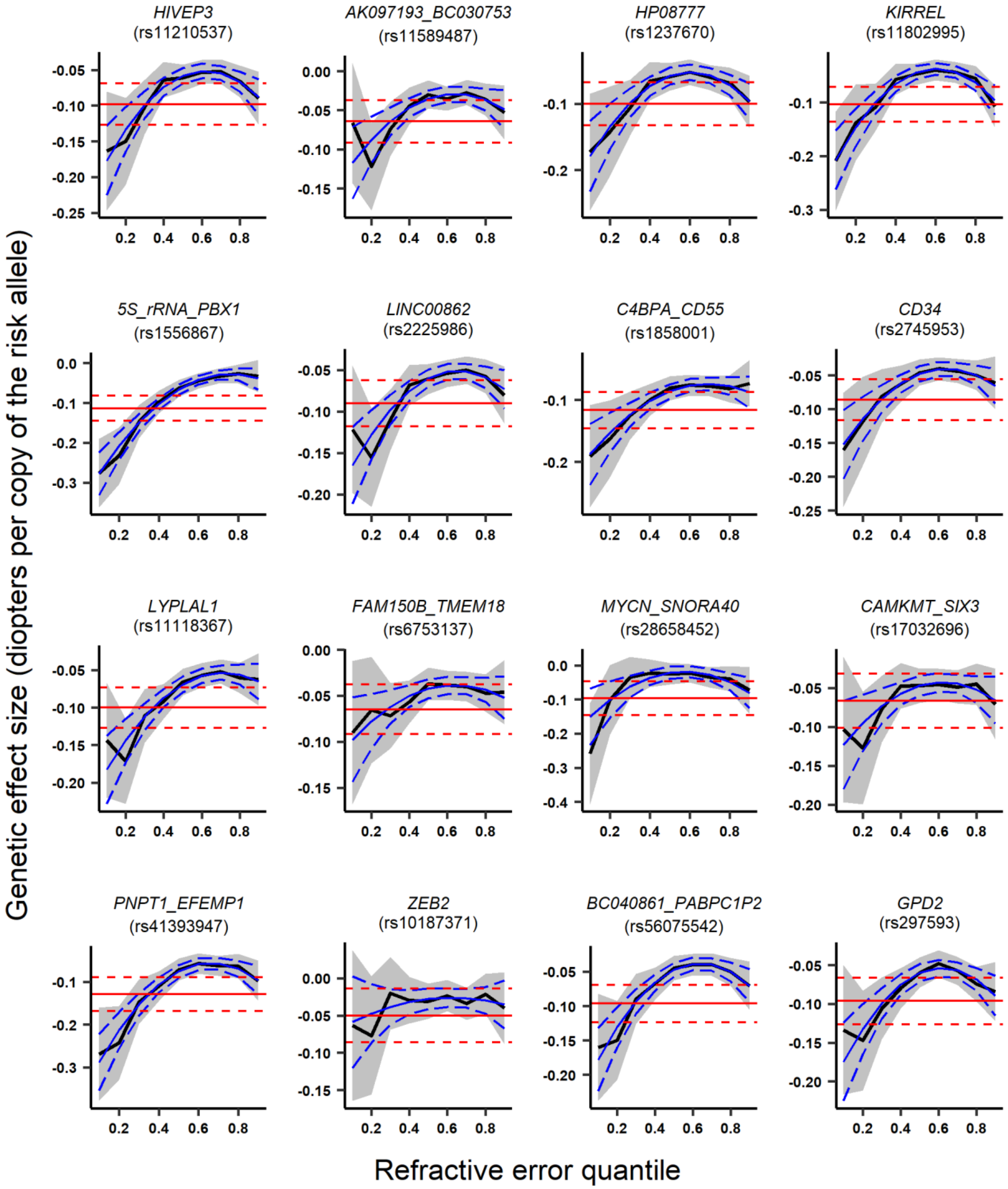
HIVEP3
(rs11210537)
AK097193_BC030753
(rs11589487)
HP08777
(rs1237670)
KIRREL
(rs11802995)
5S_rRNA_PBX1
(rs1556867)
LINC00862
(rs2225986)
C4BPA_CD55
(rs1858001)
CD34
(rs2745953)
LYPLAL1
(rs11118367)
FAM150B_TMEM18
(rs6753137)
MYCN_SNORA40
(rs28658452)
CAMKMT_SIX3
(rs17032696)
PNPT1_EFEMP1
(rs41393947)
ZEB2
(rs10187371)
BC040861_PABPC1P2
(rs56075542)
GPD2
(rs297593)
Genetic effect size (diopters per copy of the risk allele)
Refractive error quantile

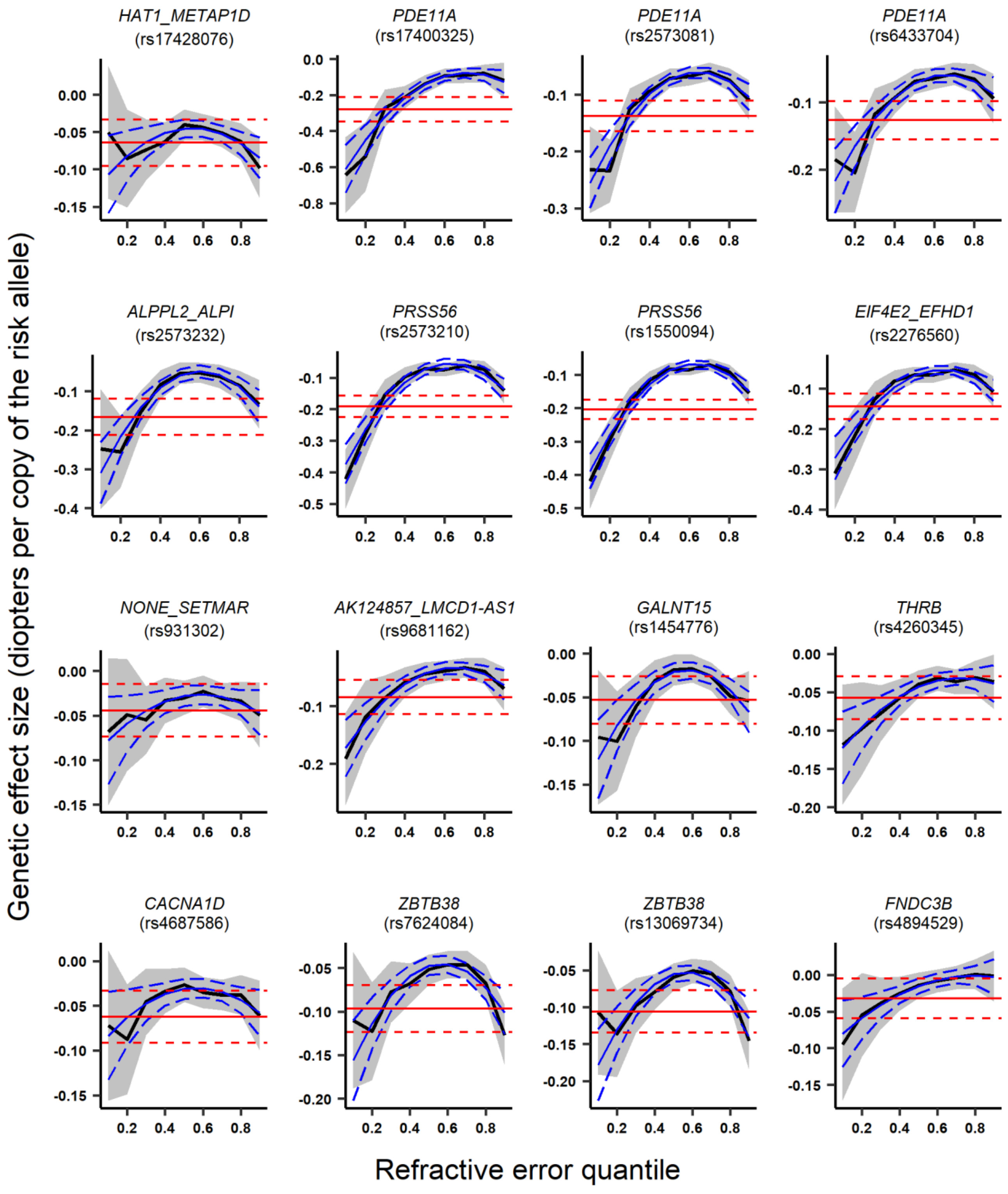

HAT1_METAP1D (rs17428076)
PDE11A (rs17400325)
PDE11A (rs2573081)
PDE11A (rs6433704)
ALPPL2_ALPI (rs2573232)
PRSS56 (rs2573210)
PRSS56 (rs1550094)
EIF4E2_EFHD1 (rs2276560)
NONE_SETMAR (rs931302)
AK124857_LMCD1-AS1 (rs9681162)
GALNT15 (rs1454776)
THRB (rs4260345)
CACNA1D (rs4687586)
ZBTB38 (rs7624084)
ZBTB38 (rs13069734)
FNDC3B (rs4894529)
Genetic effect size (diopters per copy of the risk allele)
Refractive error quantile

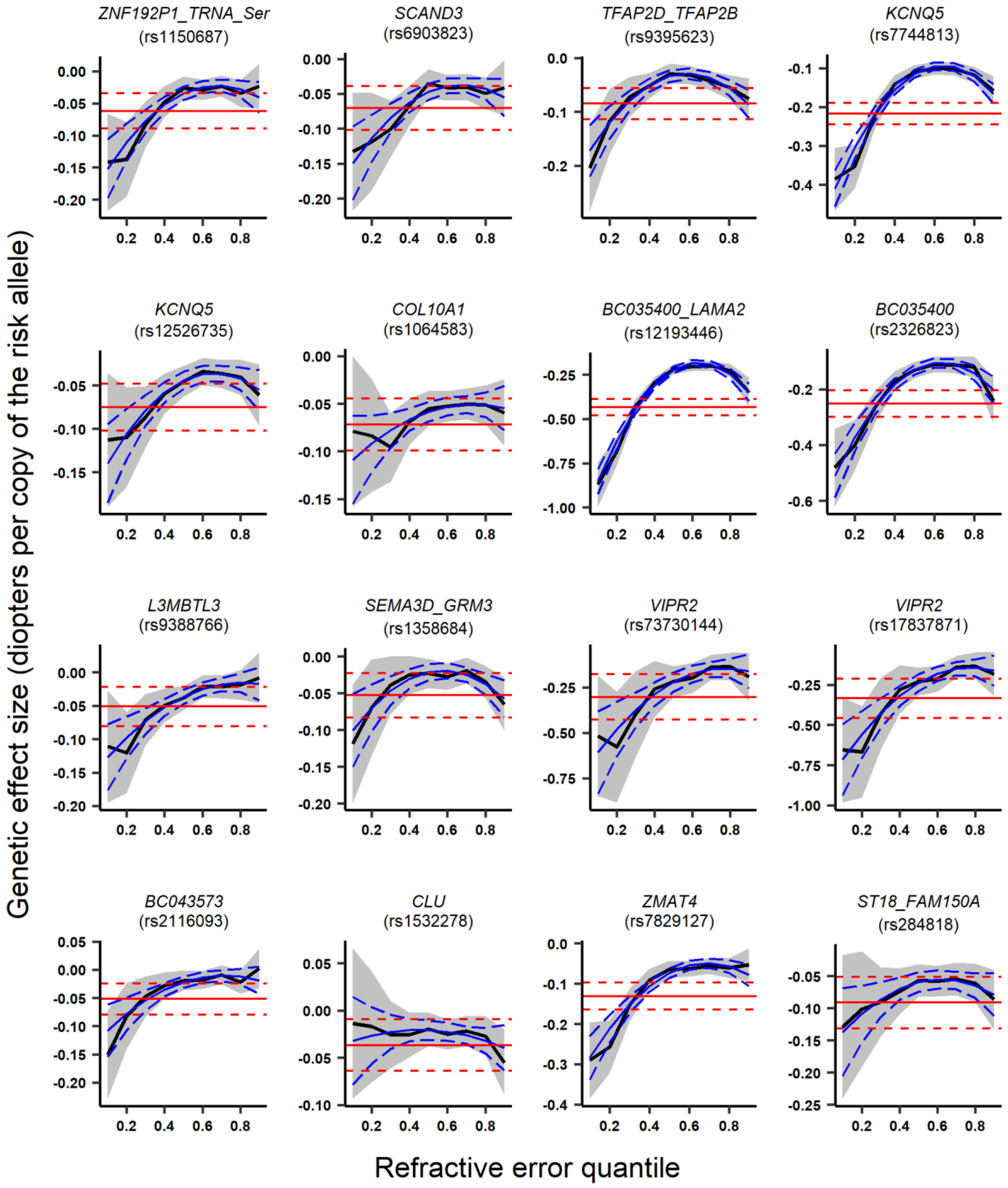
ZNF192P1_TRNA_Ser (rs1150687)
SCAND3 (rs6903823)
TFAP2D_TFAP2B (rs9395623)
KCNQ5 (rs7744813)
KCNQ5 (rs12526735)
COL10A1 (rs1064583)
BC035400_LAMA2 (rs12193446)
BC035400 (rs2326823)
L3MBTL3 (rs9388766)
SEMA3D_GRM3 (rs1358684)
VIPR2 (rs73730144)
VIPR2 (rs17837871)
BC043573 (rs2116093)
CLU (rs1532278)
ZMAT4 (rs7829127)
ST18_FAM150A (rs284818)
Genetic effect size (diopters per copy of the risk allele)
Refractive error quantile

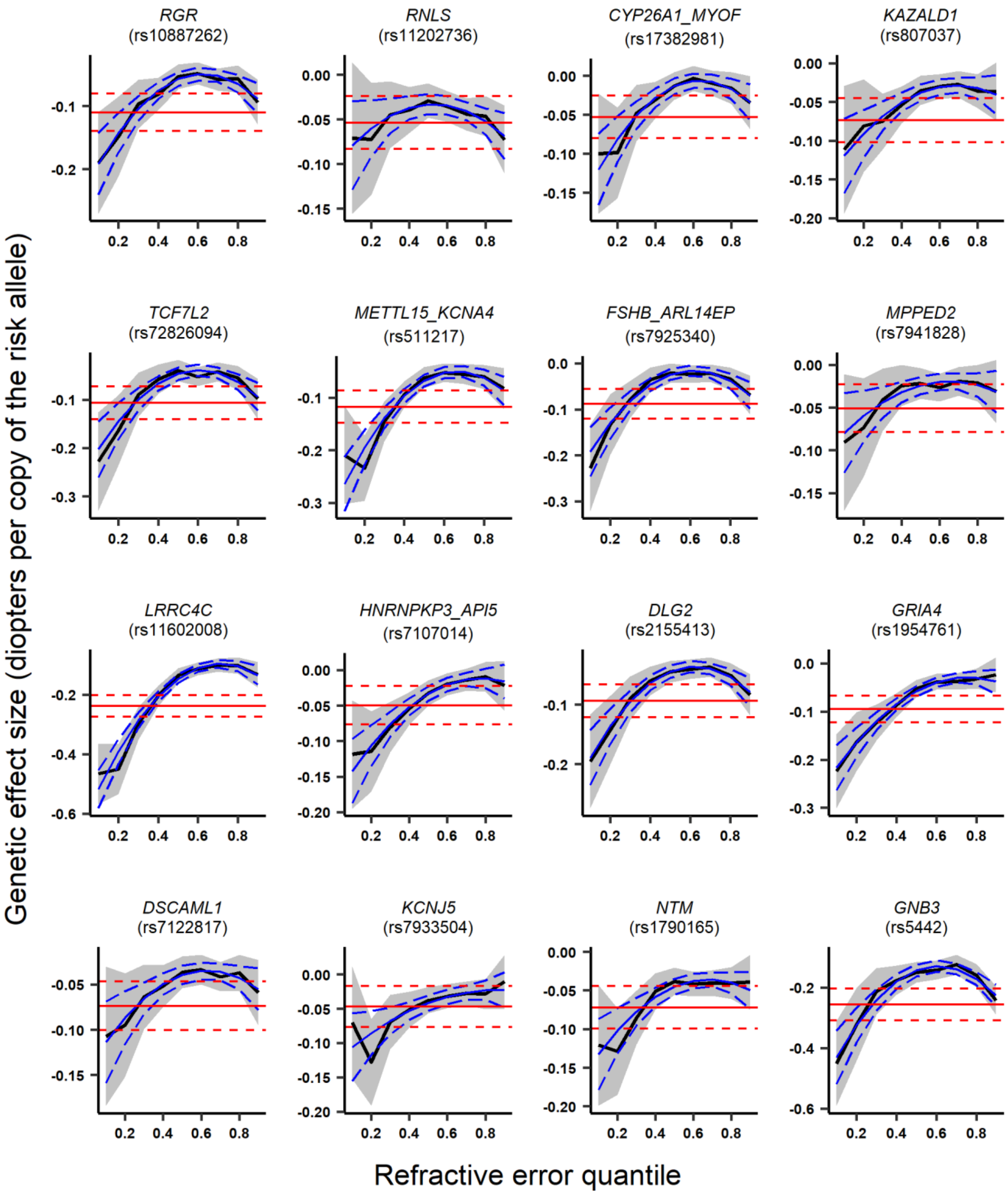
RGR
(rs10887262)
RNLS
(rs11202736)
CYP26A1_MYOF
(rs17382981)
KAZALD1
(rs807037)
TCF7L2
(rs72826094)
METTL15_KCNA4
(rs511217)
FSHB_ARL14EP
(rs7925340)
MPPED2
(rs7941828)
LRRC4C
(rs11602008)
HNRNPKP3_API5
(rs7107014)
DLG2
(rs2155413)
GRIA4
(rs1954761)
DSCAML1
(rs7122817)
KCNJ5
(rs7933504)
NTM
(rs1790165)
GNB3
(rs5442)
Genetic effect size (diopters per copy of the risk allele)
Refractive error quantile

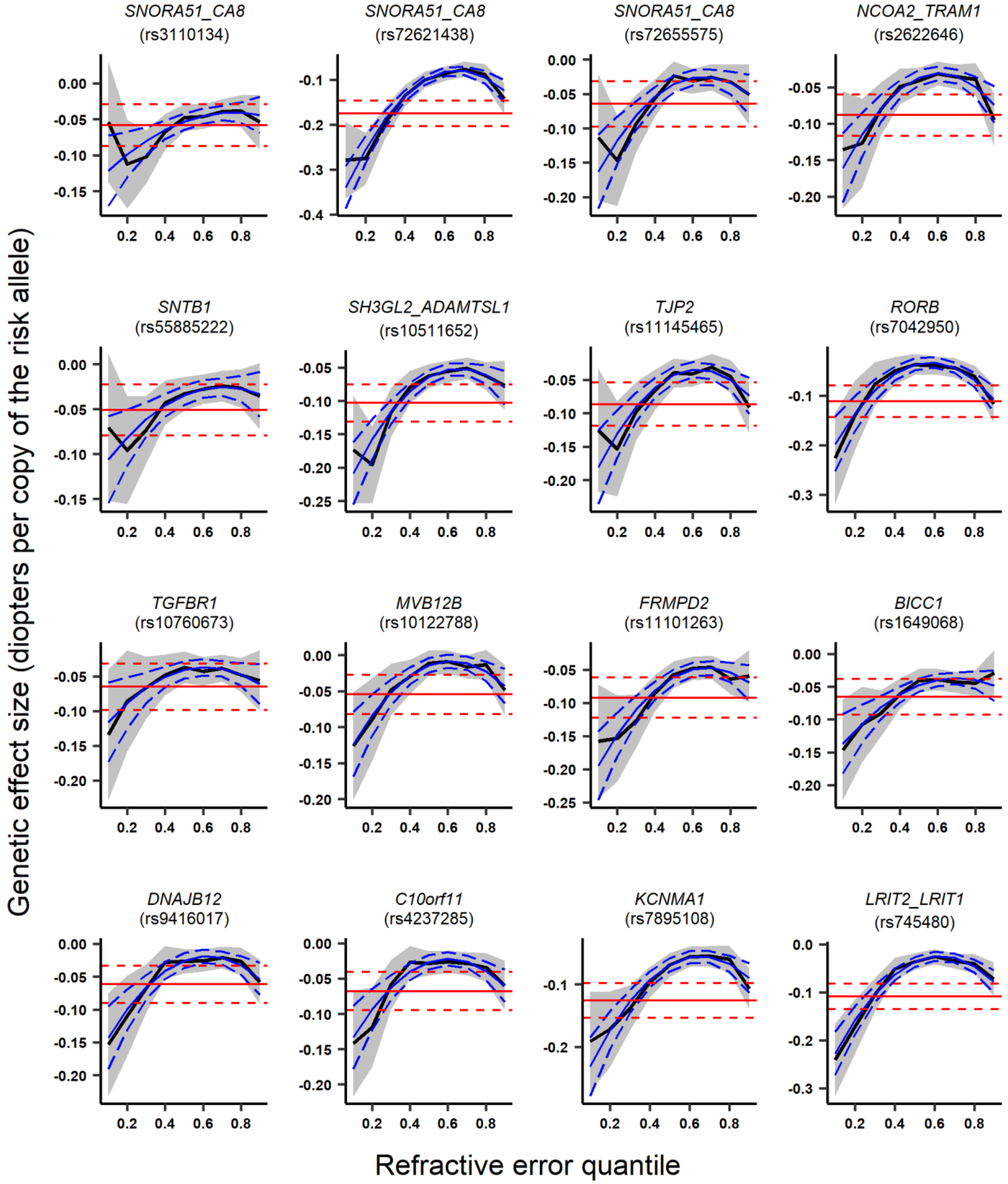
SNORA51_CA8 (rs3110134)
SNORA51_CA8 (rs72621438)
SNORA51_CA8 (rs72655575)
NCOA2_TRAM1 (rs2622646)
SNTB1 (rs55885222)
SH3GL2_ADAMTSL1 (rs10511652)
TJP2 (rs11145465)
RORB (rs7042950)
TGFBR1 (rs10760673)
MVB12B (rs10122788)
FRMPD2 (rs11101263)
BICC1 (rs1649068)
DNAJB12 (rs9416017)
C10orf11 (rs4237285)
KCNMA1 (rs7895108)
LRIT2_LRIT1 (rs745480)
Genetic effect size (diopters per copy of the risk allele)
Refractive error quantile

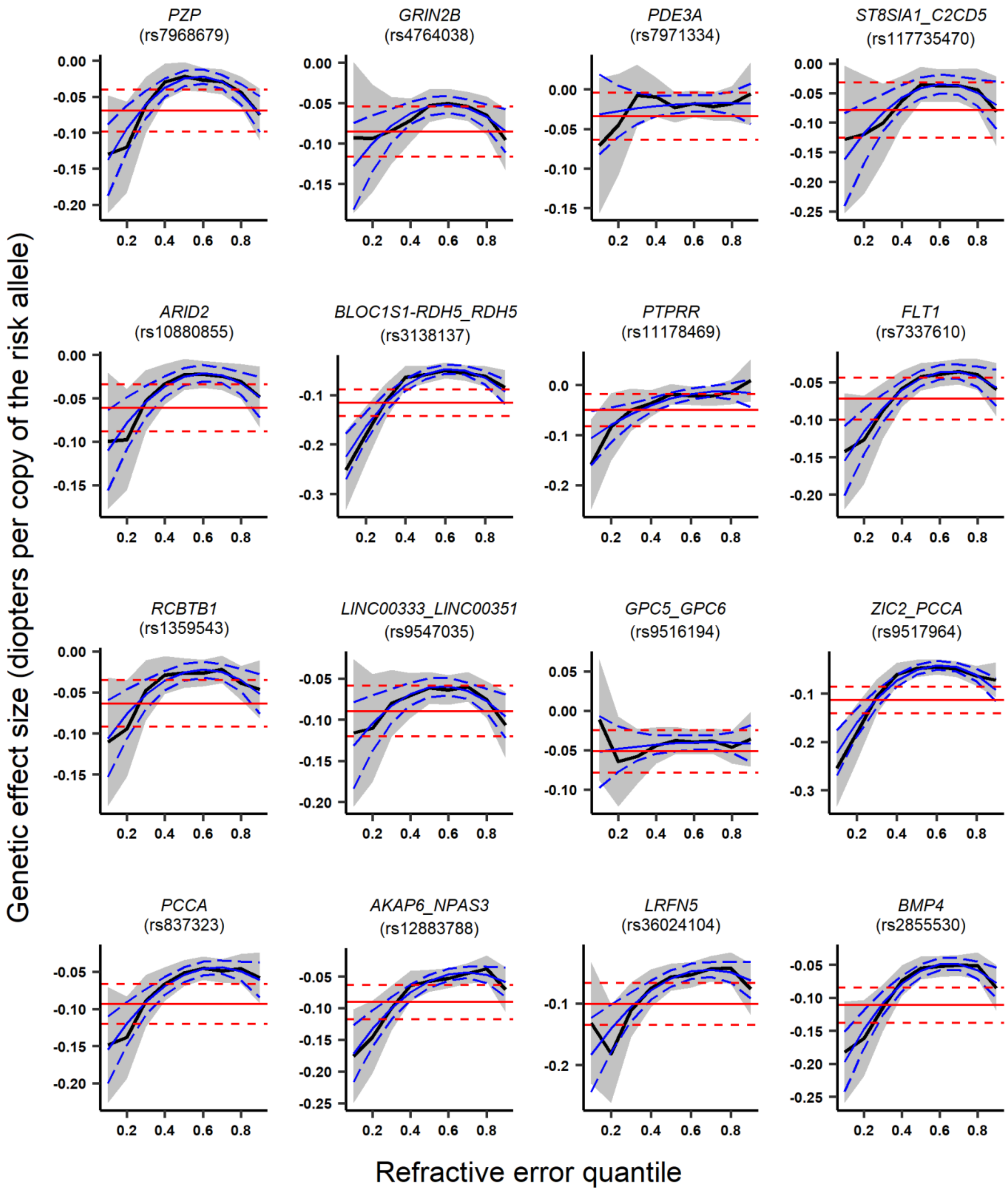

PZP
(rs7968679)
GRIN2B
(rs4764038)
PDE3A
(rs7971334)
ST8SIA1_C2CD5
(rs117735470)
ARID2
(rs10880855)
BLOC1S1-RDH5_RDH5
(rs3138137)
PTPRR
(rs11178469)
FLT1
(rs7337610)
RCBTB1
(rs1359543)
LINC00333_LINC00351
(rs9547035)
GPC5_GPC6
(rs9516194)
ZIC2_PCCA
(rs9517964)
PCCA
(rs837323)
AKAP6_NPAS3
(rs12883788)
LRFN5
(rs36024104)
BMP4
(rs2855530)
Genetic effect size (diopters per copy of the risk allele)
Refractive error quantile

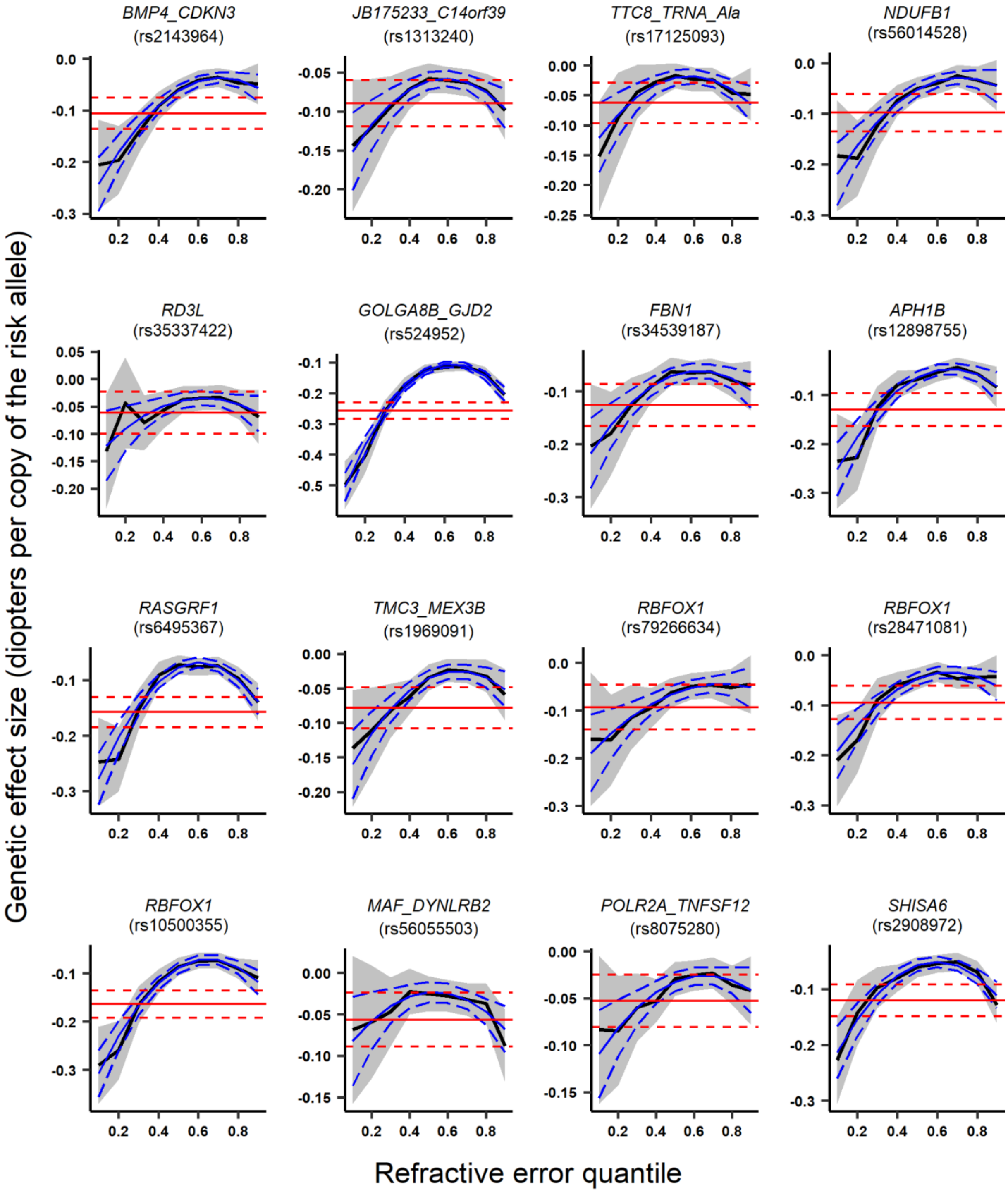
BMP4_CDKN3
(rs2143964)
JB175233_C14orf39
(rs1313240)
TTC8_TRNA_Ala
(rs17125093)
NDUFB1
(rs56014528)
RD3L
(rs35337422)
GOLGA8B_GJD2
(rs524952)
FBN1
(rs34539187)
APH1B
(rs12898755)
RASGRF1
(rs6495367)
TMC3_MEX3B
(rs1969091)
RBFOX1
(rs79266634)
RBFOX1
(rs28471081)
RBFOX1
(rs10500355)
MAF_DYNLRB2
(rs56055503)
POLR2A_TNFSF12
(rs8075280)
SHISA6
(rs2908972)
Genetic effect size (diopters per copy of the risk allele)
Refractive error quantile

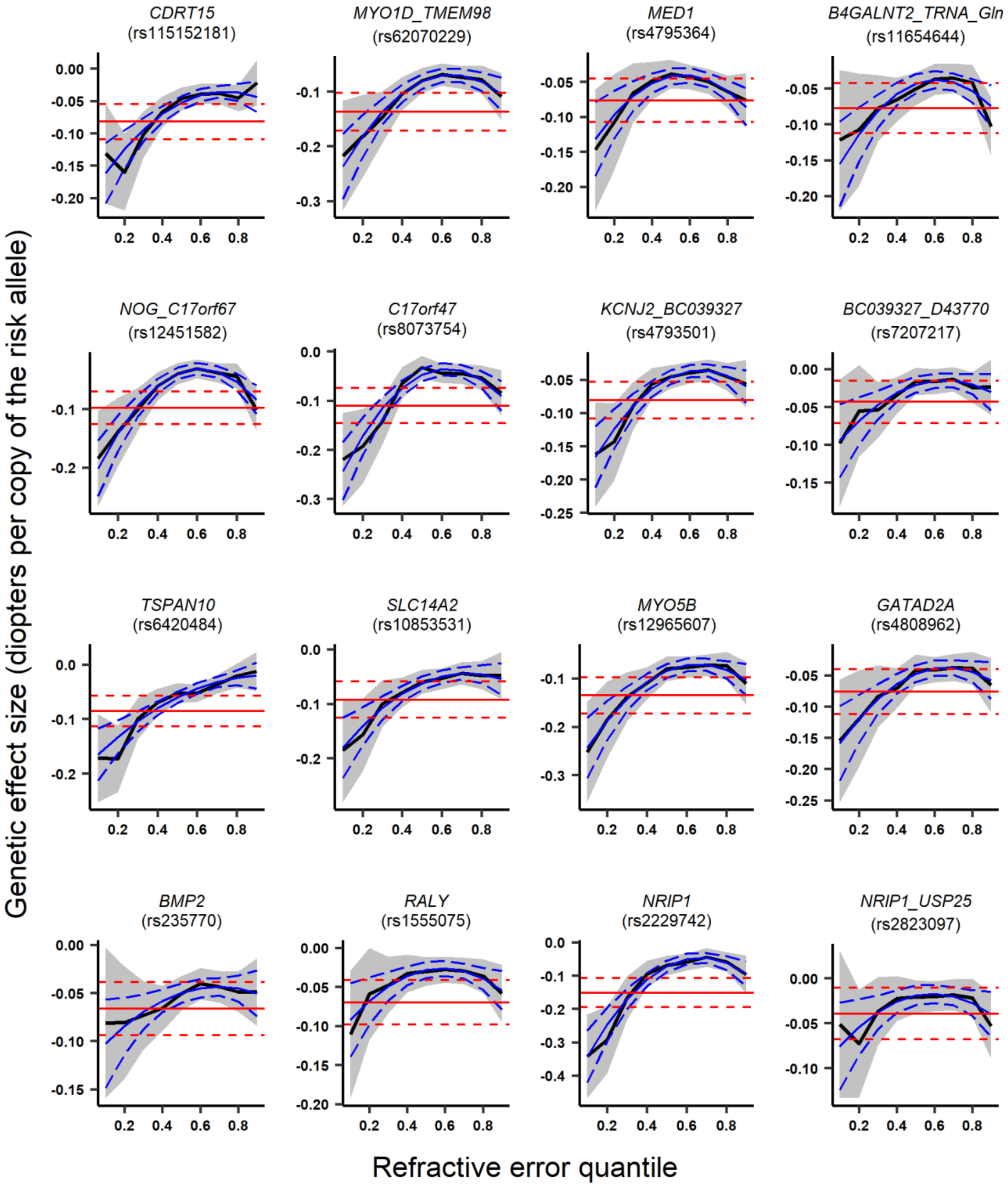
CDRT15 (rs115152181)
MYO1D_TMEM98 (rs62070229)
MED1 (rs4795364)
B4GALNT2_TRNA_Gln (rs11654644)
NOG_C17orf67 (rs12451582)
C17orf47 (rs8073754)
KCNJ2_BC039327 (rs4793501)
BC039327_D43770 (rs7207217)
TSPAN10 (rs6420484)
SLC14A2 (rs10853531)
MYO5B (rs12965607)
GATAD2A (rs4808962)
BMP2 (rs235770)
RALY (rs1555075)
NRIP1 (rs2229742)
NRIP1_USP25 (rs2823097)
Genetic effect size (diopters per copy of the risk allele)
Refractive error quantile

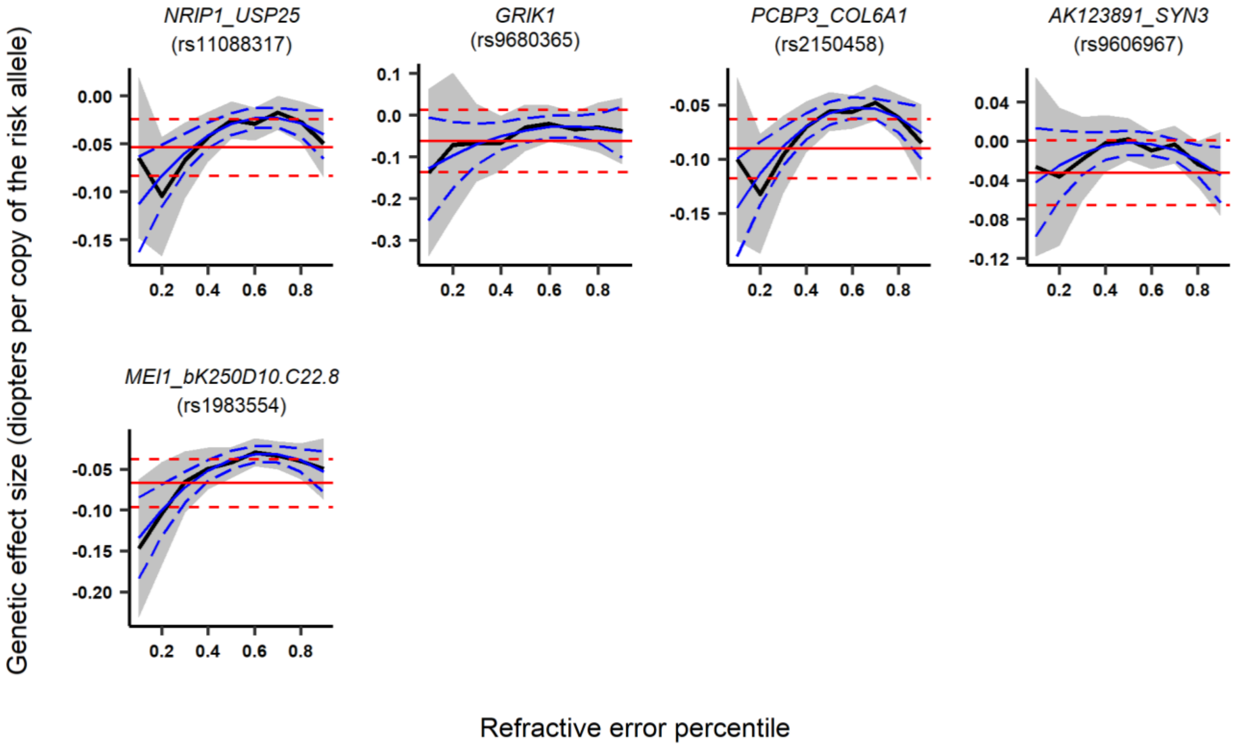

Supplementary Figure 2. Changes in genetic effect size across the refractive error distribution for genetic variants associated with refractive error. Genetic effect size estimates from conditional quantile regression (CQR) are represented by the solid black line and their 95% confidence intervals are shown by the shaded grey region. The solid red line is the effect size estimate from conventional OLS linear regression analysis with its 95% confidence intervals shown by the red dashed lines. Effect size estimates from meta-regression are shown with the solid blue line and 95% confidence intervals given by the dashed blue lines.

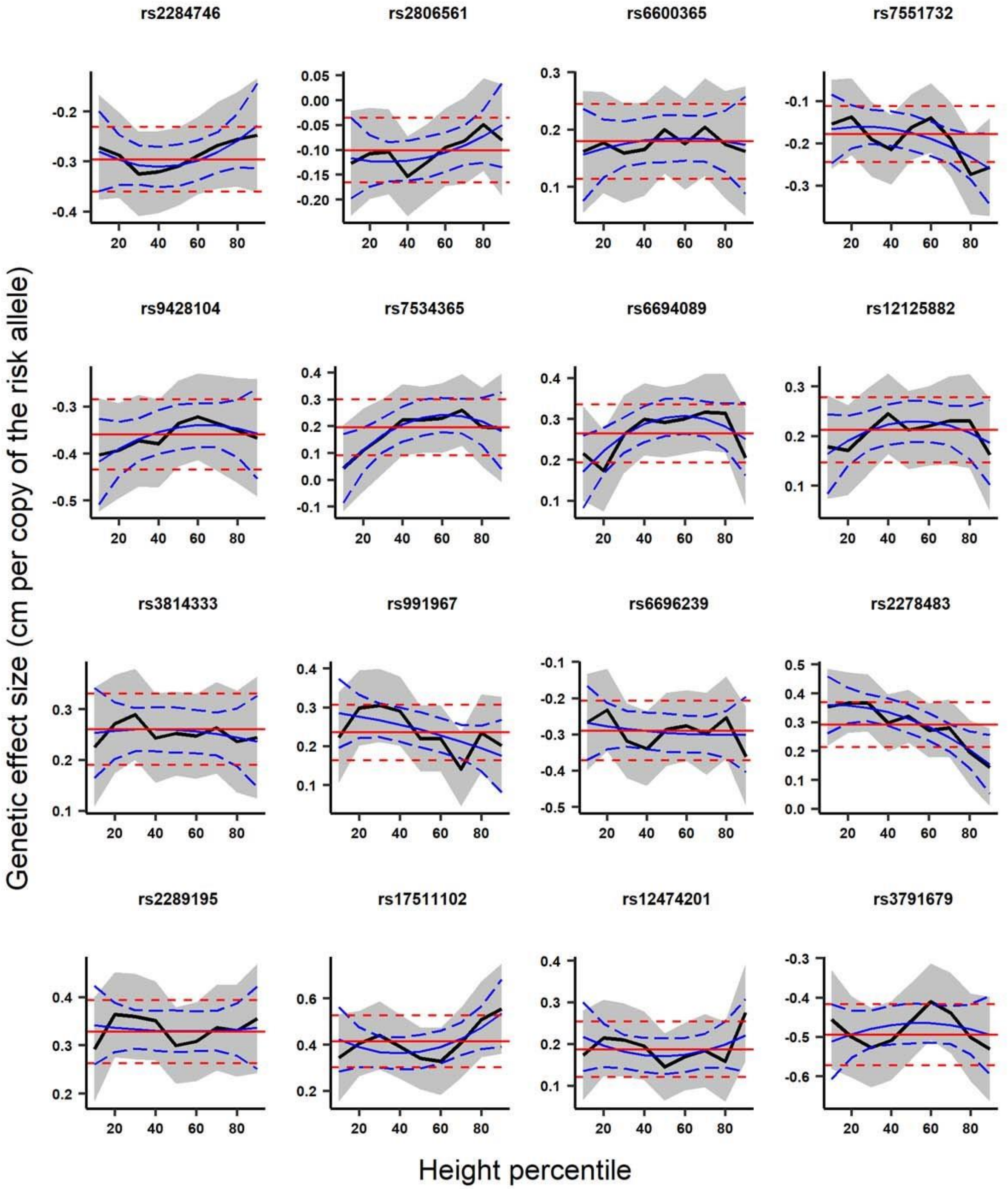
rs2284746
rs2806561
rs6600365
rs7551732
rs9428104
rs7534365
rs6694089
rs12125882
rs3814333
rs991967
rs6696239
rs2278483
rs2289195
rs17511102
rs12474201
rs3791679
Genetic effect size (cm per copy of the risk allele)
Height percentile

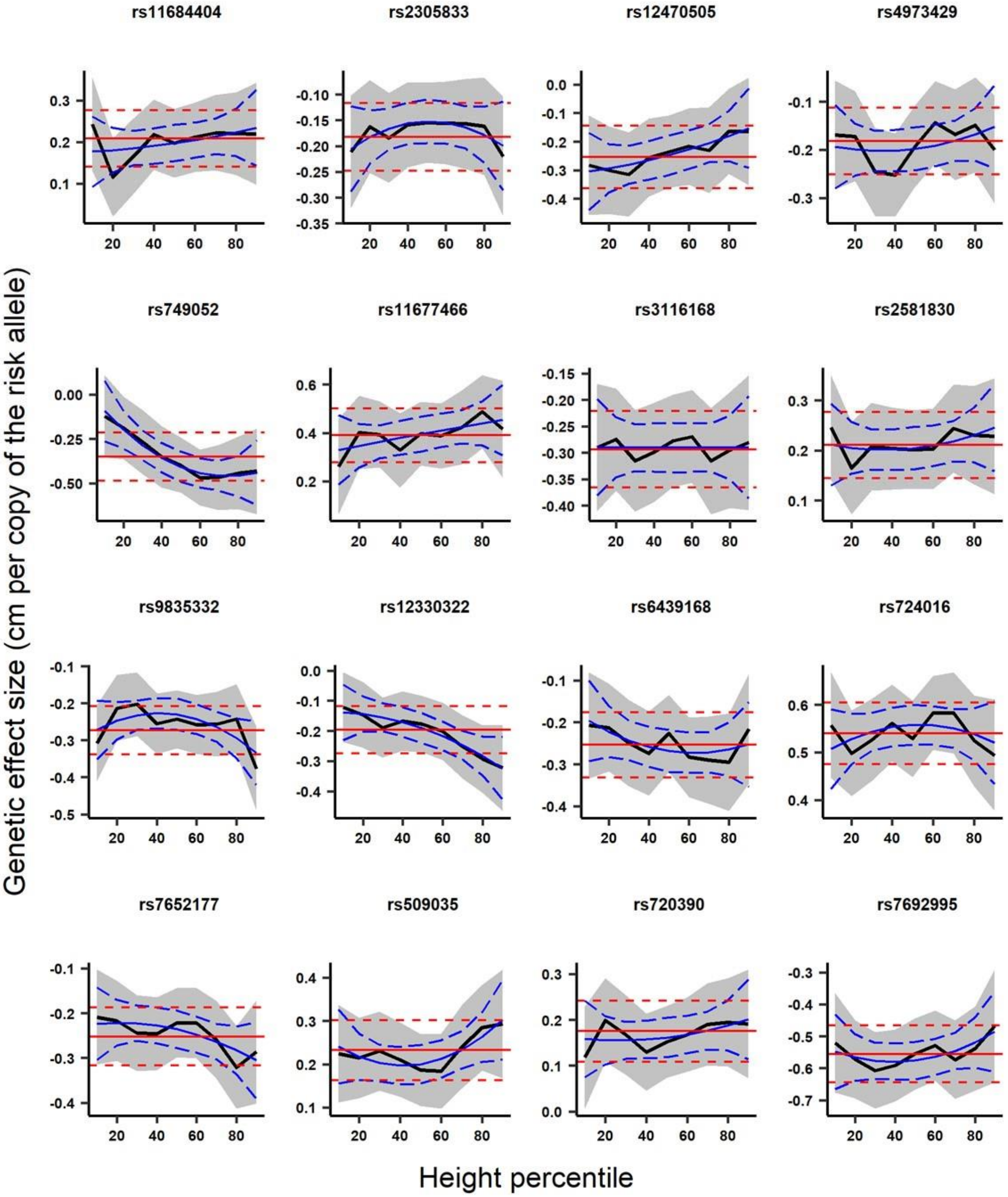
rs11684404
rs2305833
rs12470505
rs4973429
rs749052
rs11677466
rs3116168
rs2581830
rs9835332
rs12330322
rs6439168
rs724016
rs7652177
rs509035
rs720390
rs7692995
Genetic effect size (cm per copy of the risk allele)
Height percentile

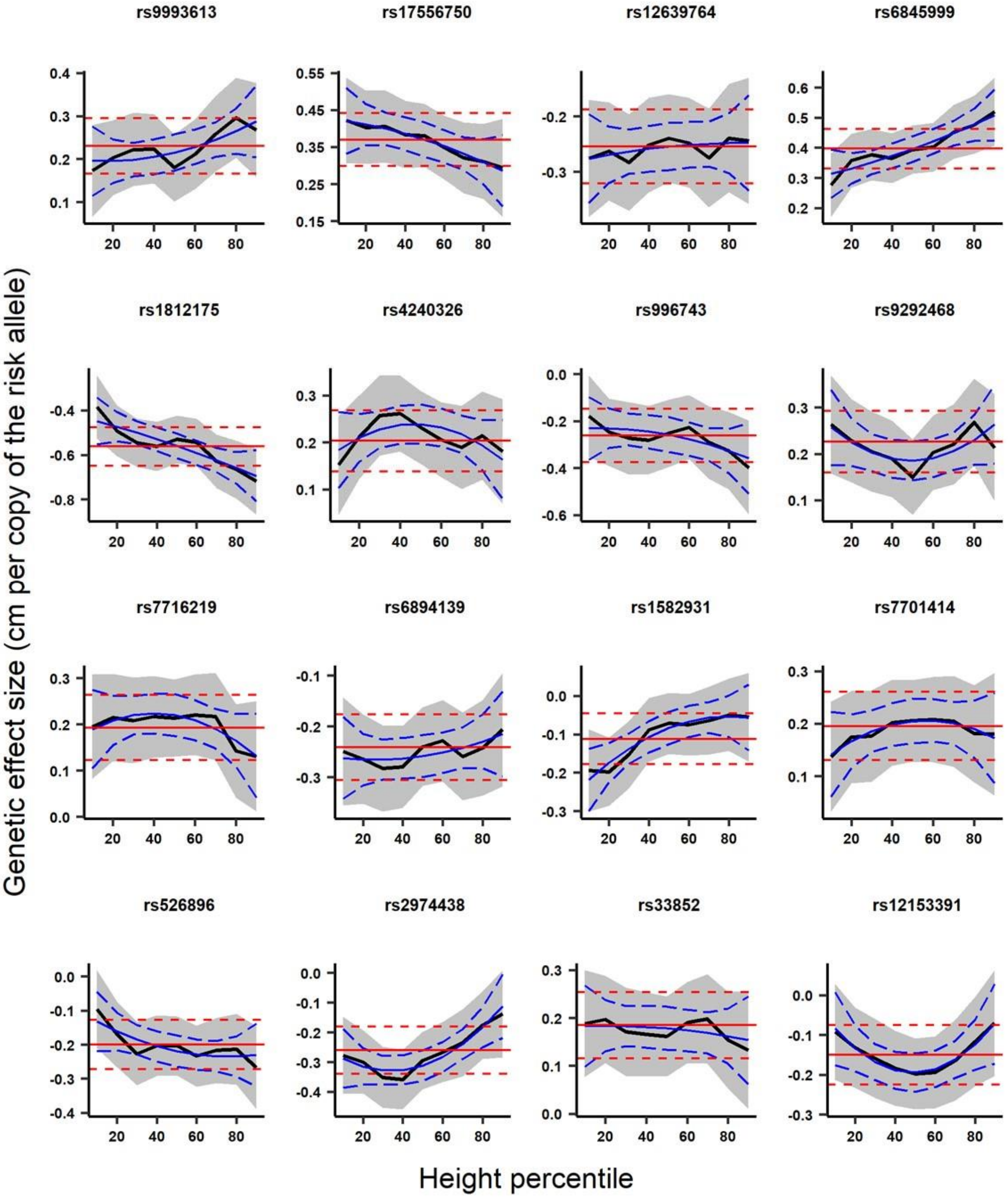

rs9993613
rs17556750
rs12639764
rs6845999
rs1812175
rs4240326
rs996743
rs9292468
rs7716219
rs6894139
rs1582931
rs7701414
rs526896
rs2974438
rs33852
rs12153391
Genetic effect size (cm per copy of the risk allele)
Height percentile

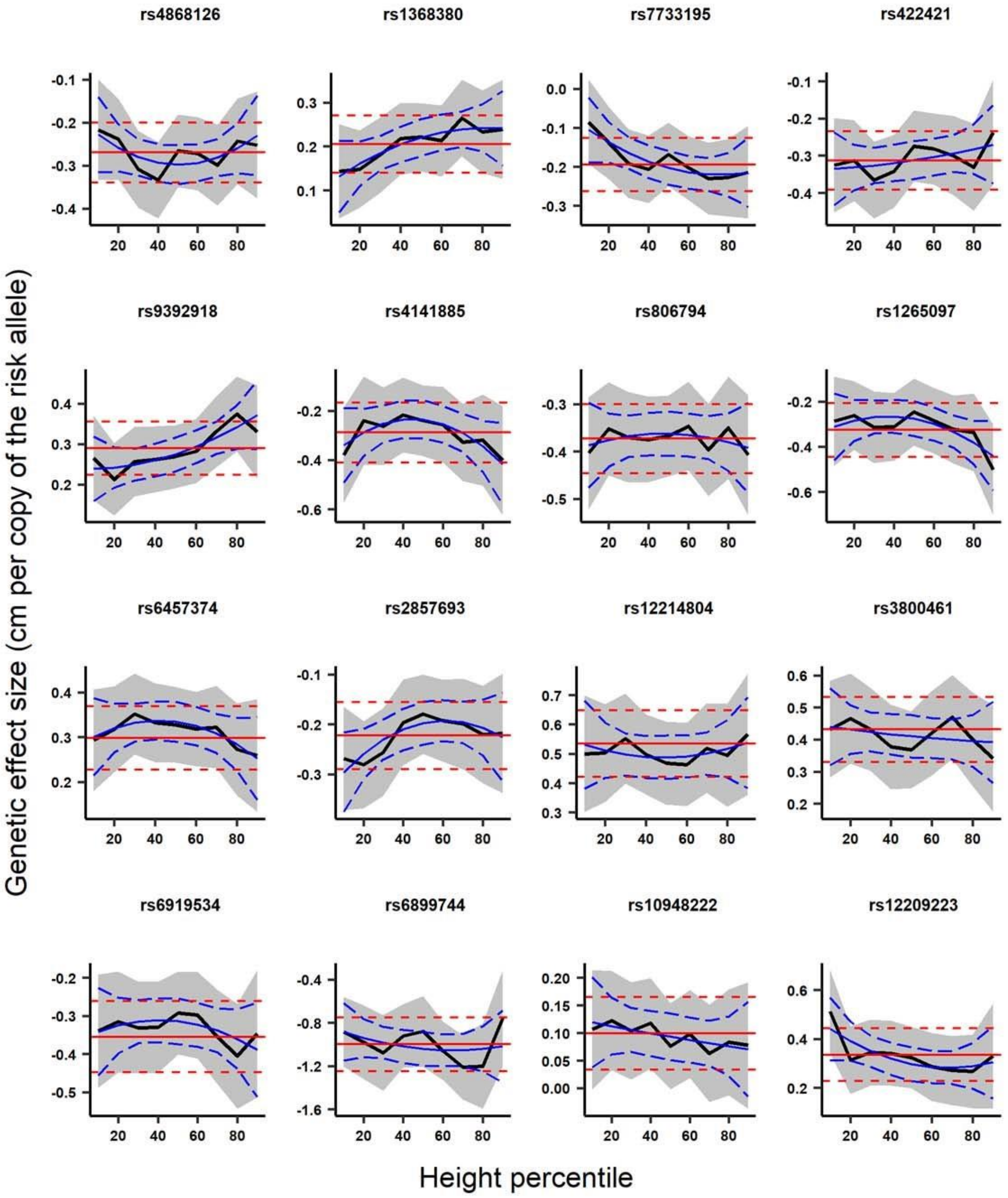
rs4868126
rs1368380
rs7733195
rs422421
rs9392918
rs4141885
rs806794
rs1265097
rs6457374
rs2857693
rs12214804
rs3800461
rs6919534
rs6899744
rs10948222
rs12209223
Genetic effect size (cm per copy of the risk allele)
Height percentile

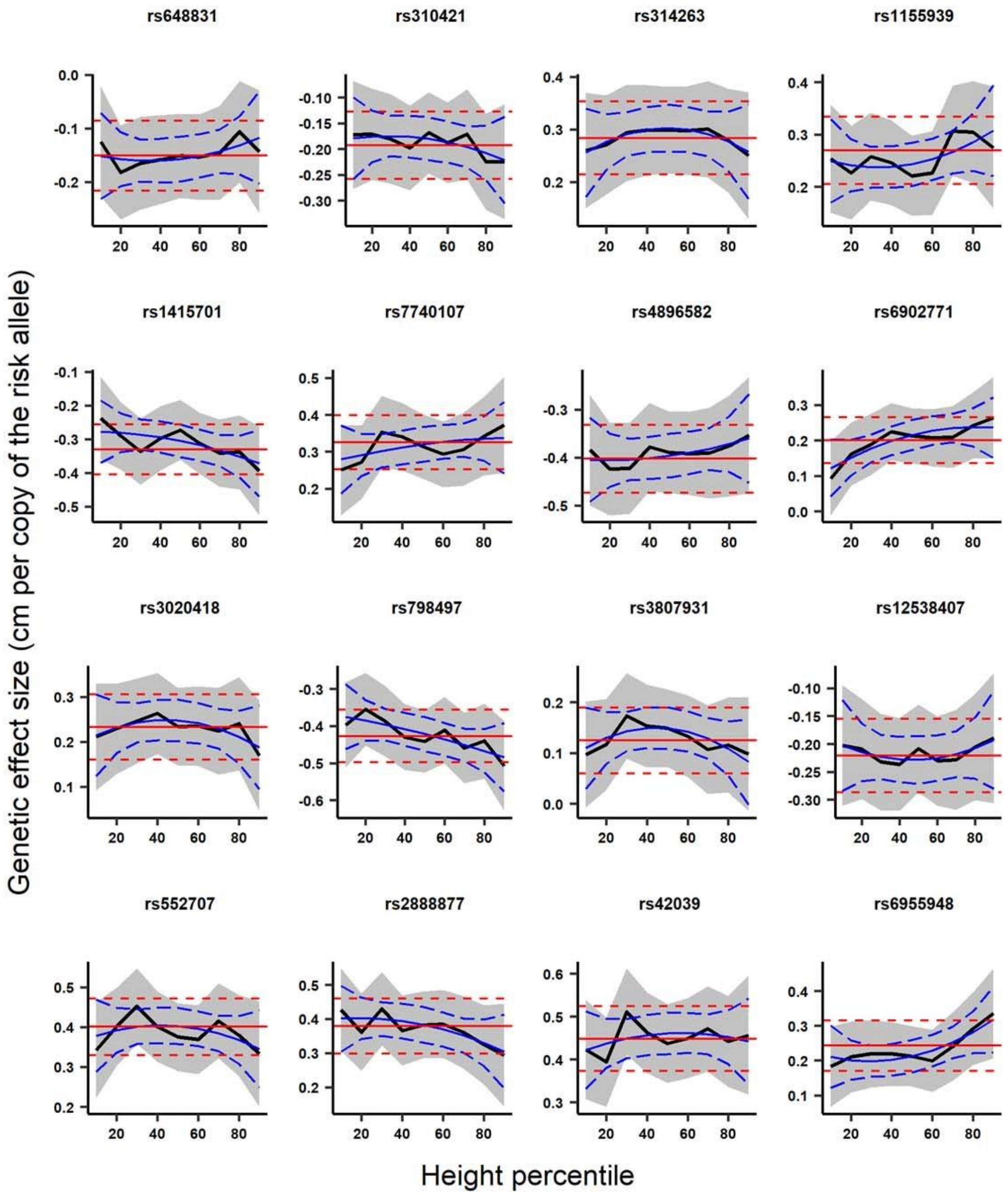
rs648831
rs310421
rs314263
rs1155939
rs1415701
rs7740107
rs4896582
rs6902771
rs3020418
rs798497
rs3807931
rs12538407
rs552707
rs2888877
rs42039
rs6955948
Genetic effect size (cm per copy of the risk allele)
Height percentile

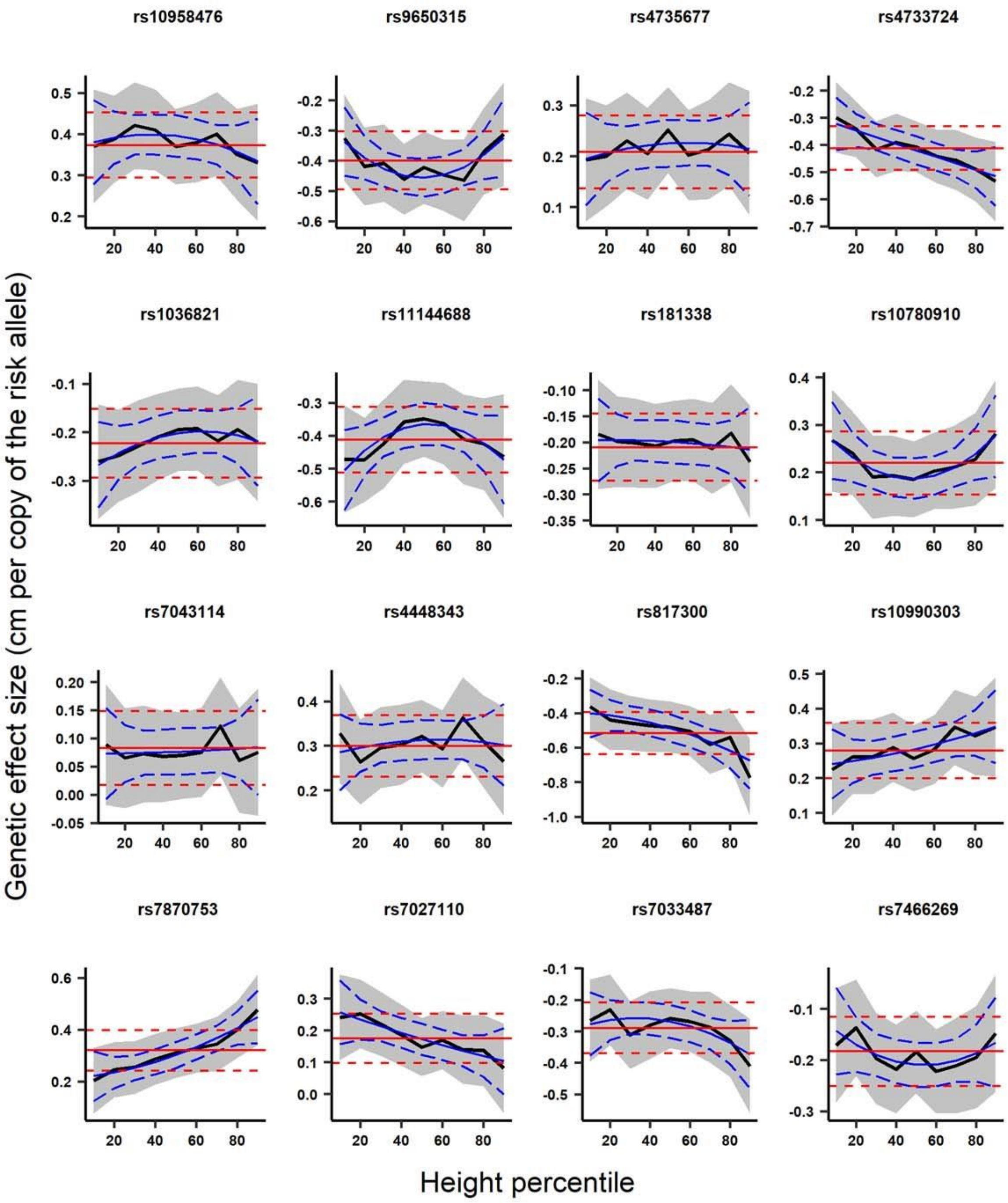

rs10958476
rs9650315
rs4735677
rs4733724
rs1036821
rs11144688
rs181338
rs10780910
rs7043114
rs4448343
rs817300
rs10990303
rs7870753
rs7027110
rs7033487
rs7466269
Genetic effect size (cm per copy of the risk allele)
Height percentile

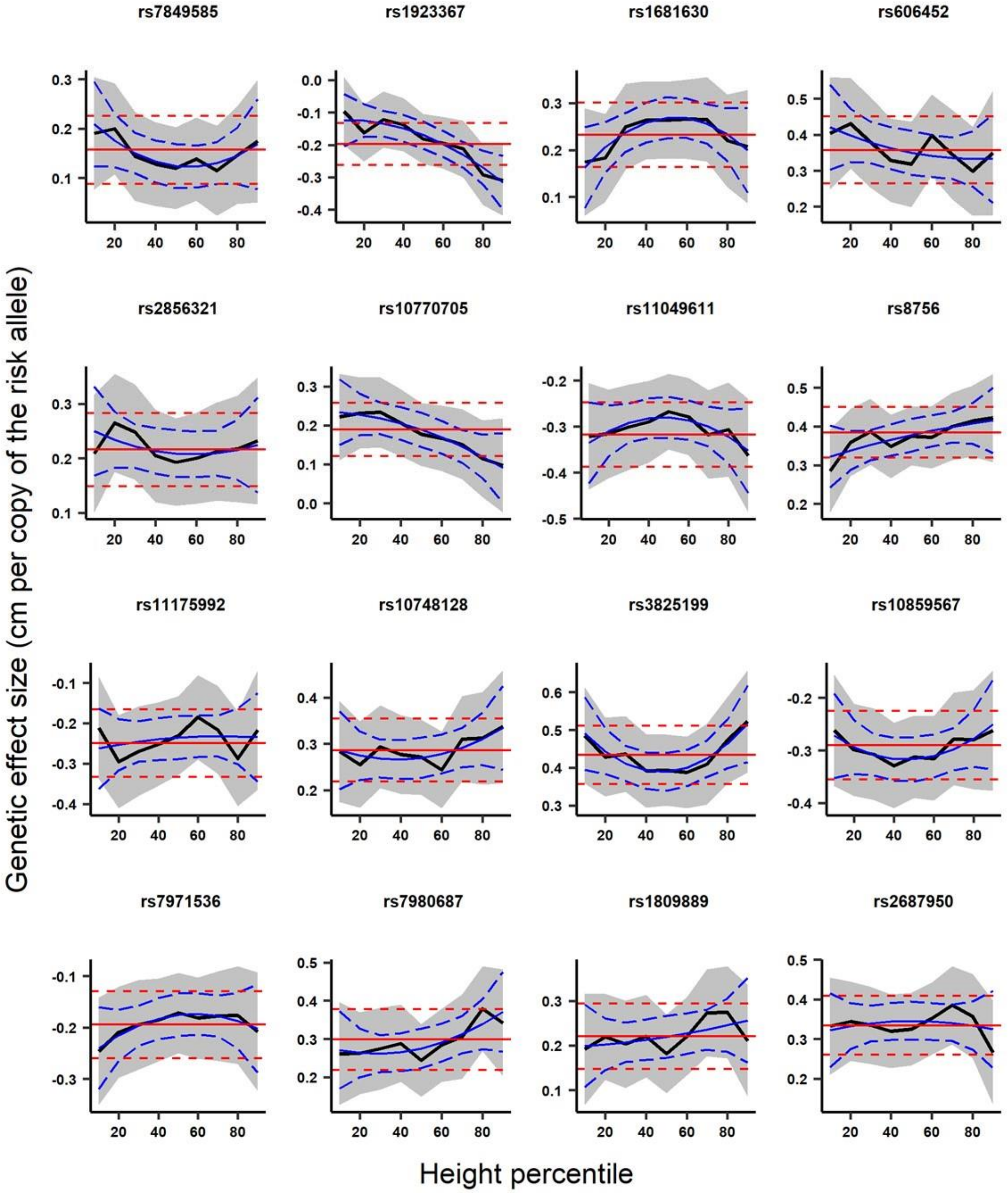
rs7849585
rs1923367
rs1681630
rs606452
rs2856321
rs10770705
rs11049611
rs8756
rs11175992
rs10748128
rs3825199
rs10859567
rs7971536
rs7980687
rs1809889
rs2687950
Genetic effect size (cm per copy of the risk allele)
Height percentile

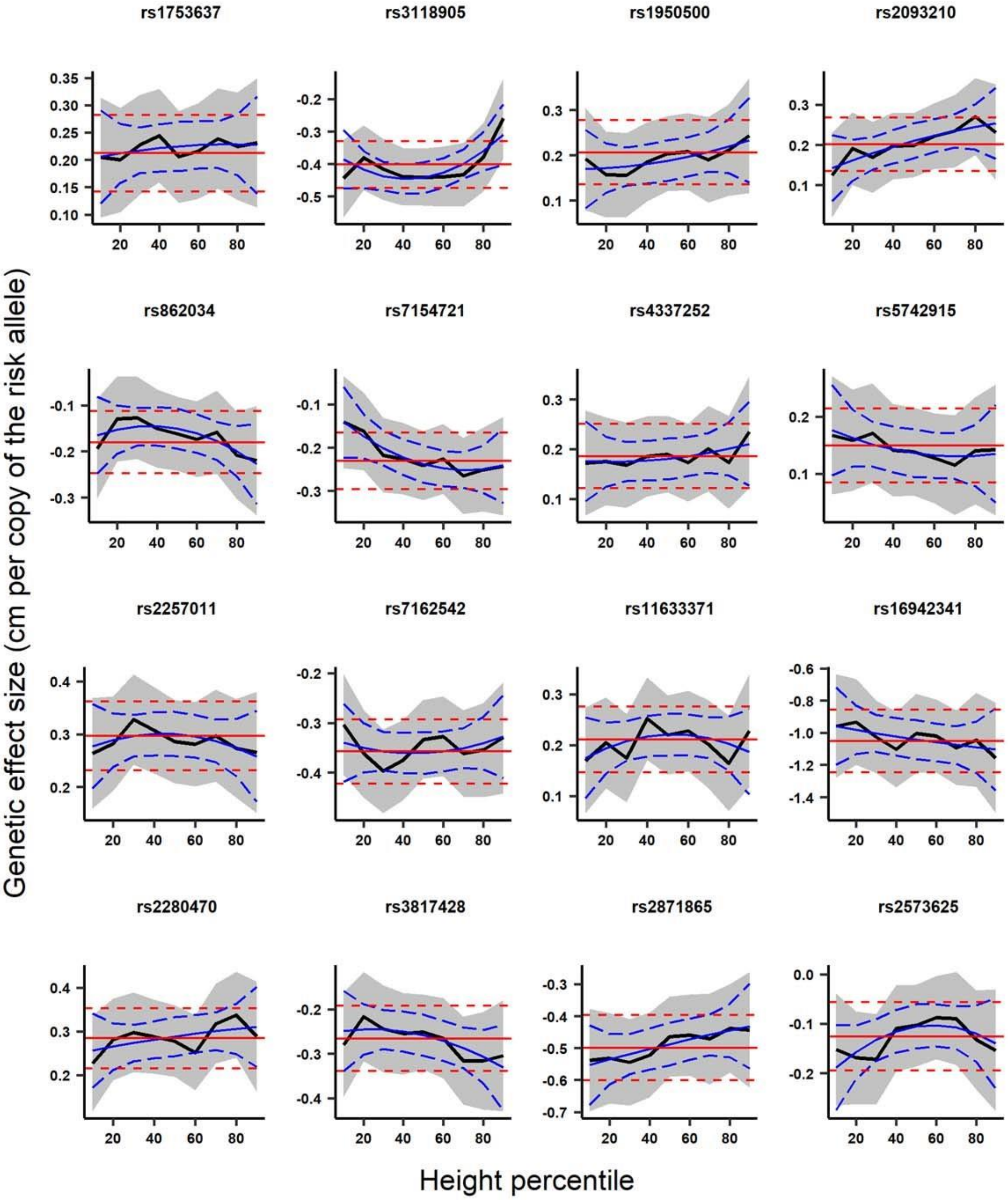
rs1753637
rs3118905
rs1950500
rs2093210
rs862034
rs7154721
rs4337252
rs5742915
rs2257011
rs7162542
rs11633371
rs16942341
rs2280470
rs3817428
rs2871865
rs2573625
Genetic effect size (cm per copy of the risk allele)
Height percentile

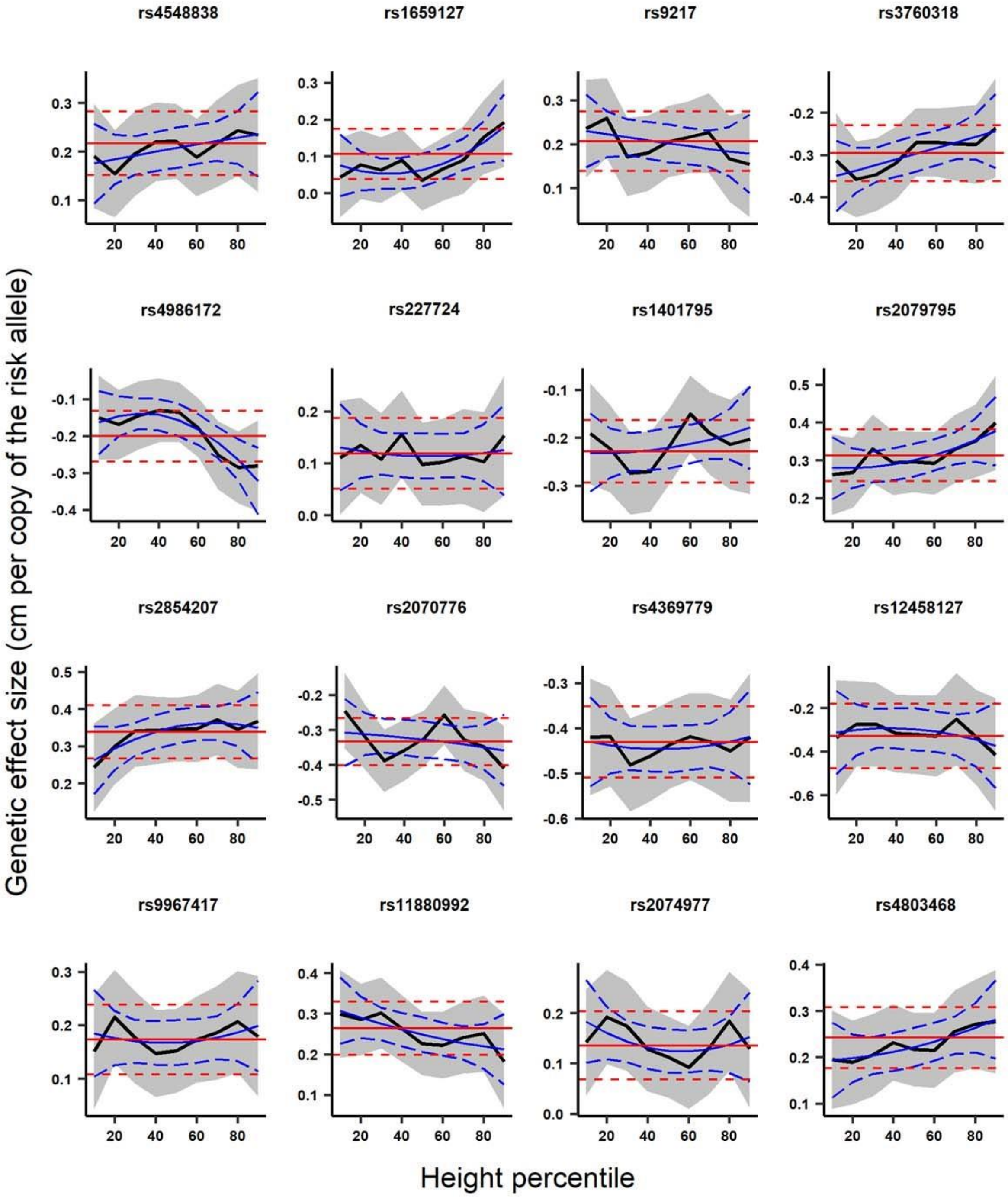
rs4548838
rs1659127
rs9217
rs3760318
rs4986172
rs227724
rs1401795
rs2079795
rs2854207
rs2070776
rs4369779
rs12458127
rs9967417
rs11880992
rs2074977
rs4803468
Genetic effect size (cm per copy of the risk allele)
Height percentile

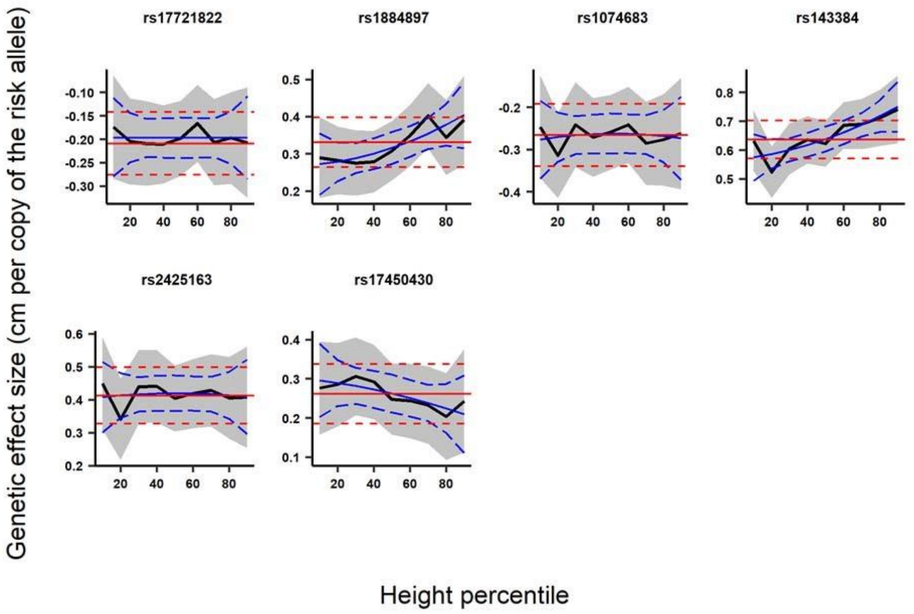

Supplementary Figure 3. Changes in genetic effect size across the height distribution for genetic variants associated with height. Summary statistics for height were obtained from Wood et al. 2014. Genetic effect size estimates from conditional quantile regression (CQR) are represented by the solid black line and their 95% confidence intervals are shown by the shaded grey region. The solid red line is the effect size estimate from conventional linear regression analysis with its 95% confidence intervals shown by the red dashed lines. Effect size estimates from meta-regression are shown with the solid blue line and 95% confidence intervals given by the dashed blue lines.

Supplementary Note 1

Type I error and power of CQR-MR

CQR is known to have a well-controlled type I error rate[3]. However, the non-linear meta-regression (MR) approach we used to combine results from across different quantiles has not been studied previously in the context of CQR to our knowledge. Therefore, we used the gold standard method of permutation to examine how the type I error rate and power of the 3 terms of our CQR-MR model (β_0, β_1 and β_2; i.e. the intercept, linear and quadratic terms) varied depending on the number of quantiles included in the MR model. As illustrated in Supplementary Figure 1, A & B, we evaluated MR models that included 5, 9, 10 or 19 quantiles from across the trait distribution. The main findings were: [1] there was a systematic inflation of the type I error rate for all 3 terms in the CQR-MR model (Supplementary Figure 1, C), [2] the type I error rate of CQR-MR was independent of MAF (Supplementary Figure 1, D), and [3] the model including 5 quantiles was overly conservative (Supplementary Figure 1, E). Fortunately, the systematic nature of this source of bias meant that it was straightforward to correct for (see below).

For the CQR-MR intercept term (β_0), inflation of the type I error rate was apparent for all models, becoming progressively worse when greater numbers of quantiles were included in the MR. For example, the model with 19 quantiles had a type I error rate of approximately 0.30, while the model with 10 quantiles had a type I error rate of 0.16. The type I error rate for the model with 5 quantiles was 0.06, which was close to – but still above – the correct type I error rate ($\alpha = 0.05$). The type I error rate for the CQR-MR linear and quadratic terms (β_1 and β_2 coefficients respectively) was also inflated for most of the CQR-MR models tested, with the degree of inflation again worsening when greater numbers of quantiles were included in the MR model (Supplementary Figure 1, C & D). However, for CQR-MR models that included only 5 quantiles, the type I error rate for the linear and quadratic terms was slightly conservative (approximately 0.04).

After correcting for the inflated type I error rate (see below), the statistical power was similar when either 9, 10 or 19 quantiles were included in the CQR-MR model, however power was reduced when only 5 quantiles were included in the MR model (Supplementary Figure 1, E).

Thus, CQR-MR models that included 9, 10 or 19 quantiles had inflated type I error rates for all 3 terms in the model, requiring the use of correction factors to account for this bias. An MR model that included only 5 quantiles had a conservative type I error rate, and was less powerful than MR models that included 9, 10 or 19 quantiles. Hence, we selected a CQR-MR model that included 9 quantiles as the optimal model. To correct the type I error rate, the intercept, linear and quadratic components of the model were adjusted using λ coefficients of 1.66, 1.23 and 1.10, respectively (i.e. observed Chi-squared statistics were divided by the relevant λ coefficient when calculating confidence intervals and p-values).

Supplementary Note 2

Adjustment for the inflated type 1 error rate of CQR-MR

Meta-regression was found to produce a systematically inflated type 1 error rate (see above). To adjust for this source of bias, we calculated 'inflation factors' ($\lambda\beta_0$, $\lambda\beta_1$ and $\lambda\beta_2$) analogous to the use of λ_{GC} for genomic control[4], using the results from the 'null phenotype' permutation analyses described in the Methods section. P-values and confidence intervals for each term (β_0, β_1 and β_2) in the meta-regression were adjusted by their respective inflation factor with the equation: $X^2_{adjusted} = X^2_{observed}/\lambda$, where λ was calculated as the observed median chi-squared statistic from the 'null phenotype' permutations divided by the expected median chi-squared statistic with 1 df. Noting that Z-statistic $= \beta/s.e.$ and $X^2 = Z^2$, meta-regression confidence intervals were calculated by adjusting standard errors: $s.e._{\text{adjusted}} = |\beta / \sqrt{X^2_{adjusted}}|$.

Supplementary Note 3

CQR-MR analysis of GIANT consortium variants associated with height

For comparison with refractive error, we performed an analogous CQR-MR analysis of height – a trait previously shown to have a limited extent of GxG or GxE interaction[1,2]. We selected the 149 variants most strongly associated with height identified by the GIANT consortium[2]. One variant (rs6899744) was removed because of low MAF (2%), leaving 148 variants. In the OLS linear regression analyses, the G allele of rs143384 located near the *GDF5* gene had both the largest effect size and the strongest association with height (effect size = +0.64 cm, 95% CI 0.57 to 0.70, $p = 2.14 \times 10^{-80}$). In the quantile regression analyses, most variants had uniform effects across height quantiles; those that did not generally exhibited more linear profiles as compared to those for refractive error (Supplementary Figure 2), with the strongest effect occurring at either quantile 0.05 (e.g. *CENPO* variant rs2278483 and *STAU1* variant rs17450430) or quantile 0.95 (e.g. *HHIP* variant rs1812175 and *FAM46A* variant rs310421). Results for all variants are presented in Supplementary Figure 2.

After applying a permutation-based correction to control the type-I error rate of the CQR-MR analyses for height, 53% of the variants exhibited an association with height (meta-regression intercept $p < 3.34 \times 10^{-4}$), of which the largest effect was observed for rs143384 located near the GDF5 gene: intercept $\beta_0 = 0.53$ (95% CI 0.45 to 0.62), $p = 1.14 \times 10^{-17}$. However, none of the 148 variants exhibited evidence of a non-uniform effect size across the sample distribution (Bonferroni adjusted *p*-value threshold $0.05/(3 \times 148) = 3.34 \times 10^{-4}$; Supplementary Table 4). Indeed, of the 148 GIANT consortium variants tested, 139 (94%) failed to show even nominal evidence of an interaction effect (i.e. β_1 component and β_2 component, $p > 0.05$ after correcting for inflation of the type-I error rate of the meta-regression analysis).

Supplementary References

1 Abadi, A. et al. Penetrance of Polygenic Obesity Susceptibility Loci across the Body Mass Index Distribution. Am. J. Hum. Genet. 101, 925-938 (2017).

2 Wood, A. R. et al. Defining the role of common variation in the genomic and biological architecture of adult human height. Nat. Genet. 46, 1173-1186 (2014).

3 Koenker, R. & Hallock, K. F. Quantile Regression. Journal of Economic Perspectives 15, 143-156 (2001).

4 Devlin, B. & Roeder, K. Genomic control for association studies. Biometrics 55, 997-1004 (1999).
