# Supplementary material for: Quantile regression analysis reveals widespread evidence for gene-environment or gene-gene interactions in myopia development
Source: Commun Biol. 2019 May 6;2:167. doi: 10.1038/s42003-019-0387-5 (PMC6502837; doi:10.1038/s42003-019-0387-5)
Supplement: Supplementary file 2 — Description of additional supplementary items [file 42003_2019_387_MOESM2_ESM.docx]

**Description of additional supplementary items**

**Supplementary Data 1.** Summary statistics for ‘conventional’ OLS regression effect size estimates for association with refractive error.

**Supplementary Data 2.** Summary statistics for CQR-MR effect estimates for variants associated with refractive error.

**Supplementary Data 3.** Summary statistics for ‘conventional’ OLS regression effect size estimates for association with height.

**Supplementary Data 4.** Summary statistics for CQR-MR effect estimates for variants associated with height.
