# Supplementary material for: Quantile regression analysis reveals widespread evidence for gene-environment or gene-gene interactions in myopia development
Source: Commun Biol. 2019 May 6;2:167. doi: 10.1038/s42003-019-0387-5 (PMC6502837; doi:10.1038/s42003-019-0387-5)
Supplement: Supplementary file 4 — Reporting Summary [file 42003_2019_387_MOESM4_ESM.pdf]

nature research

Corresponding author(s): Jeremy A. Guggenheim

Last updated by author(s): Jan 8, 2019

Reporting Summary

Nature Research wishes to improve the reproducibility of the work that we publish. This form provides structure for consistency and transparency in reporting. For further information on Nature Research policies, see Authors & Referees and the Editorial Policy Checklist.

Statistics

For all statistical analyses, confirm that the following items are present in the figure legend, table legend, main text, or Methods section.

n/a	Confirmed	
☐	☒	The exact sample size (*n*) for each experimental group/condition, given as a discrete number and unit of measurement
☒	☐	A statement on whether measurements were taken from distinct samples or whether the same sample was measured repeatedly
☐	☒	The statistical test(s) used AND whether they are one- or two-sided<br>*Only common tests should be described solely by name; describe more complex techniques in the Methods section.*
☐	☒	A description of all covariates tested
☐	☒	A description of any assumptions or corrections, such as tests of normality and adjustment for multiple comparisons
☐	☒	A full description of the statistical parameters including central tendency (e.g. means) or other basic estimates (e.g. regression coefficient) AND variation (e.g. standard deviation) or associated estimates of uncertainty (e.g. confidence intervals)
☐	☒	For null hypothesis testing, the test statistic (e.g. *F*, *t*, *r*) with confidence intervals, effect sizes, degrees of freedom and *P* value noted<br>*Give P values as exact values whenever suitable.*
☒	☐	For Bayesian analysis, information on the choice of priors and Markov chain Monte Carlo settings
☒	☐	For hierarchical and complex designs, identification of the appropriate level for tests and full reporting of outcomes
☐	☒	Estimates of effect sizes (e.g. Cohen's *d*, Pearson's *r*), indicating how they were calculated

Our web collection on statistics for biologists contains articles on many of the points above.

Software and code

Policy information about availability of computer code

Data collection	N/A
Data analysis	R version 3.5.1 (packages: quantreg v5.36; metafor v2.0.0)

For manuscripts utilizing custom algorithms or software that are central to the research but not yet described in published literature, software must be made available to editors/reviewers. We strongly encourage code deposition in a community repository (e.g. GitHub). See the Nature Research guidelines for submitting code & software for further information.

Data

Policy information about availability of data

All manuscripts must include a data availability statement. This statement should provide the following information, where applicable:

- Accession codes, unique identifiers, or web links for publicly available datasets
- A list of figures that have associated raw data
- A description of any restrictions on data availability

We will add a data availability statement at the next opportunity.

Field-specific reporting

Please select the one below that is the best fit for your research. If you are not sure, read the appropriate sections before making your selection.

☒ Life sciences ☐ Behavioural & social sciences ☐ Ecological, evolutionary & environmental sciences

For a reference copy of the document with all sections, see nature.com/documents/nr-reporting-summary-flat.pdf

Life sciences study design

All studies must disclose on these points even when the disclosure is negative.

Sample size	Number of UK Biobank participants with data available.
Data exclusions	1) Non-European genetic ancestry; 2) Did not undergo autorefraction at UK Biobank assessment centre; 3) Self-report or hospital in-patient data indicating a condition that may affect refractive error (see list of conditions in Methods section).
Replication	No replication sample is available worldwide, hence we had to rely on applying a stringent, experiment-wide multiple-testing correction.
Randomization	N/A (observational research)
Blinding	N/A (observational research)

Reporting for specific materials, systems and methods

We require information from authors about some types of materials, experimental systems and methods used in many studies. Here, indicate whether each material, system or method listed is relevant to your study. If you are not sure if a list item applies to your research, read the appropriate section before selecting a response.

Materials & experimental systems

n/a	Involved in the study
☒	☐ Antibodies
☒	☐ Eukaryotic cell lines
☒	☐ Palaeontology
☒	☐ Animals and other organisms
☐	☒ Human research participants
☐	☒ Clinical data

Methods

n/a	Involved in the study
☒	☐ ChIP-seq
☒	☐ Flow cytometry
☒	☐ MRI-based neuroimaging

Human research participants

Policy information about studies involving human research participants

Population characteristics	UK residents aged 40-69 years old; 54% female. Full demographic details can be found in Table 2 of Cumberland et al. PLoS ONE. 2015;10(10):e0139780.
Recruitment	Full details of UK Biobank recruitment are available: Sudlow et al. PLoS Med. 2015;12(3):e1001779.
Ethics oversight	Ethical approval for the study was obtained from the National Health Service National Research Ethics Service (Ref 11/NW/0382).

Note that full information on the approval of the study protocol must also be provided in the manuscript.

Clinical data

Policy information about clinical studies

All manuscripts should comply with the ICMJE guidelines for publication of clinical research and a completed CONSORT checklist must be included with all submissions.

Clinical trial registration	N/A (Observational study)
Study protocol	Study protocols are available on the UK Biobank website: https://www.ukbiobank.ac.uk/
Data collection	See: Sudlow et al. PLoS Med. 2015;12(3):e1001779.
Outcomes	Refractive error was measured using non-cycloplegic autorefraction (Tomey RC5000; Tomey GmbH Europe, Erlangen-Tennenlohe, Germany). The mean spherical equivalent refractive error was calculated as the sphere power plus half the cylinder power, and averaged between the two eyes.
